# ﻿Morphological and phylogenetic analyses reveal two new species of the *Fusariumfujikuroi* (Hypocreales, Nectriaceae) species complex in China

**DOI:** 10.3897/mycokeys.112.133472

**Published:** 2025-01-16

**Authors:** Mingwei Zhang, Cheng Peng, Shuji Li, Chengming Tian

**Affiliations:** 1 The Key Laboratory for Silviculture and Conservation of the Ministry of Education, Beijing Forestry University, Beijing 100083, China Beijing Forestry University Beijing China

**Keywords:** Ascomycota, morphology, phylogeny, taxonomy

## Abstract

The *Fusariumfujikuroi* species complex (FFSC) encompasses a diverse array of more than 80 phylogenetic species with both phytopathological and clinical importance. A stable taxonomy is crucial for species in the FFSC due to their economical relevance. Fungal strains used in this study were obtained from *Castaneamollissima* and *Rubuslambertianus*, collected from Beijing and Shaanxi Province. We employ morphological and phylogenetic analyses based on partial gene fragments of the translation elongation factor 1-alpha (*tef1*), beta-tubulin (*tub2*), calmodulin (*CaM*), RNA polymerase largest subunit (*rpb1*), and RNA polymerase II second largest subunit (*rpb2*), as well as the pairwise homoplasy index tests. Studies have shown that these phylospecies are clustered in the Asian clade of the FFSC. The present study delineates two novel *Fusarium* species within the FFSC, named *F.castaneophilum* and *F.rubicola*, complemented by illustrations and descriptions.

## ﻿Introduction

*Fusarium* (Ascomycota, Hypocreales, Nectriaceae) is a large and diverse genus that includes approximately 400 recognized species found in various habitats worldwide (https://www.fusarium.org). It is also the fourth-highest-cited genus of fungi (Bhunjun et al. 2024), underscoring its widespread significance across various sectors. As one of the most economically destructive and diverse groups of mycotoxin-producing pathogens, *Fusarium* poses significant threats to plant, animal, and human health, as well as to global food security (Desjardins 2006; Leslie and Summerell 2006; O’Donnell et al. 2013; Renev et al. 2021). *Fusarium* species cause a range of diseases that impact forestry worldwide (Yilmaz et al. 2021). For example, *F.oxysporum* causes wilt of *Albiziajulibrissin*, leading to substantial damage in scenic areas where *A.julibrissin* is used as a landscape tree. In *Heveabrasiliensis*, *F.decemcellulare* causes basal rot, while *F.venfricosum* is responsible for brown spot disease; both diseases severely weaken the trees, reducing rubber production (Jiang et al. 2015). *Fusariumannulatum*, *F.avenaceum*, *F.sporotrichioides* and *F.tricinctum* cause xylem browning in *Maluspumila*, which can lead to yield reductions and potentially plant death (Cheng 2019).

The *Fusariumfujikuroi* species complex (FFSC) is one of the largest and most extensively studied species complexes within the genus (Sandoval-Denis et al. 2018a, b; Al-Hatmi et al. 2019). Species within the FFSC have been extensively researched due to their ability to cause plant infections, such as rice bakanae, maize ear rot, soybean root rot, and cankers in *Pinus* species, which lead to significant decreases in crop yield and economic income (Herron et al. 2015; O’Donnell et al. 2015; Qiu et al. 2020). FFSC was first established by Wollenweber et al. (1925) as the section Liseola, characterised by sporodochial conidia (macroconidia), microconidia in chains and/or false heads, and does not produce chlamydospores. However, later studies identified species conforming to these traits but also capable of producing chlamydospores—such as *F.beomiforme* (Nelson et al. 1987), *F.dlamini* (Marasas et al. 1985), *F.napiforme* (Marasas et al. 1987) and *F.nygamai* (Burgess and Trimboli 1986). Both *F.dlamini* and *F.beomiforme* did not produce tandem microconidia. In order to accommodate these species, Kwasna et al. (1991) introduced the section Dlaminia. Subsequent molecular studies, nonetheless, have shown that based on phylogenetic analyses of DNA sequences obtained from multiple unlinked loci, sections *Liseola* and *Dlaminia* are non-monophyletic (O’Donnell and Cigelnik 1997; O’Donnell et al. 1998a). In cases where morphology often disagreed with DNA sequence data, this highlights the challenges of using phenotypic traits to deduce relationships and evolutionary histories. With these drawbacks in mind, the term “species complex” was introduced, mainly referring to phylogenetic clades (O’Donnell and Cigelnik 1997; O’Donnell et al. 1998a).

O’Donnell et al. (1998a) developed the FFSC biogeography hypothesis and clustered the isolates into three relatively well-supported phylogenetic clades, named African, American, and Asian clades, believed to be a result of the fragmentation of Gondwana during the Upper Cretaceous through to the Paleocene. However, the authors later reported that this complex appeared much later. This apparent biogeographic aggregation may be attributed to long-distance dispersal from South America to Africa and then to Asia in the late Miocene (O’Donnell et al. 2013). In the subsequent study, the non-monophyletic African Clade was divided into two distinct and highly supported lineages: the African Clade A (core African Clade) and African Clade B (Herron et al. 2015; Sandoval-Denis et al. 2018b), along with a monotypic clade that joined the sister clades of America and Africa B, which is termed African C. Therefore, the analysis of the current dataset mainly supports five significant clades in the FFSC (Crous et al. 2021). With advancements in molecular techniques, further studies have continued to refine our understanding of FFSC’s diversity and complexity.

Presently, with the application of modern classification methods, more than 60 species have been identified in the FFSC (Yilmaz et al. 2021). Given the considerable economic impact of *Fusarium*, ongoing research is essential to uncover additional hidden species. In this study, through a combination of morphology and multi-locus phylogeny, we introduce two new species and advance our knowledge of the species diversity of fusarioid taxa from China.

## ﻿Materials and methods

### ﻿Sample collection and fungal isolation

Fungal strains used in this study were obtained from *Castaneamollissima* and *Rubuslambertianus*, collected from Beijing city and Shaanxi Province. The collected samples were placed in paper bags and transported to the laboratory for isolation. The sample surface was disinfected with 75% alcohol for 30 seconds, then with 1.25% sodium hypochlorite (NaOCl) for 90 seconds, followed by being rinsed three times with sterile water and dried on sterile filter paper. Plant tissue pieces were excised from the leaves and branches of the plant samples into 0.5 × 0.5 cm sections using a sterile blade. These plant tissue pieces were then placed on potato dextrose agar plates (PDA; containing 200 g potatoes, 20 g dextrose and 20 g agar per liter). The petri plates were cultured for three days at 25 °C in the dark. The fungal isolates were purified using the monosporic isolation method as described by Li et al. (2007). Holotype specimens of the new species identified in this study were stored at the Forest Pathology Laboratory at Beijing Forestry University and all cultures were preserved at the China Forestry Culture Collection Center (**CFCC**), Chinese Academy of Forestry, Beijing, China.

### ﻿DNA extraction, PCR and sequencing

Genomic DNAs were extracted from fungal mycelia grown on potato dextrose agar (PDA) using the cetyltrimethylammonium bromide (CTAB) method (Guo et al. 2000) and stored at -20 °C until polymerase chain reaction (PCR). Five different loci were targeted for sequencing, including the partial translation elongation factor (*tef1*), partial RNA polymerase largest subunit (*rpb1*), partial RNA polymerase second largest subunit (*rpb2*) gene regions, partial β-tubulin (*tub2*), and partial calmodulin (*CaM*), which were amplified and sequenced, respectively. The primer pairs and PCR amplification procedures are listed in Table 1. PCR amplifications were performed in a reaction mixture consisting of 10 μL 2 × ES × Taq PCR Master Mix (Tsingke Biotechnology Co. Ltd., Beijing, China), 1 μL each of primer pairs, 1 μL of undiluted genomic DNA, adjusted to a final volume of 20 μL with distilled deionized water. The PCR products were assayed by electrophoresis in 2% agarose gels. Amplified PCR products were sent to a commercial sequencing provider (Tsingke Biotechnology Co. Ltd., Beijing, China). Newly generated sequences were submitted to GenBank, with accession numbers provided in Table 2.

**Table 1.** Primer pairs, PCR amplification procedures and references used in this study.

Gene/DNA regions	Primers				PCR amplification procedures	References
Name	Abbreviation	Name	Direction	Sequence (5’→3’)^1^		
translation elongation factor 1-alpha	*tef1*	EF-1	Forward	ATGGGTAAGGARGACAAGAC	95 °C 5 min; 35 cycles of 95 °C 45 s, 55 °C 30 s, 72 °C 45 s; 72 °C 10 min; 4 °C soak	O’Donnell et al. (1998b)
		EF-2	Reverse	GGARGTACCAGTSATCATG		
RNA polymerase largest subunit	*rpb1*	Fa	Forward	CAYAARGARTCYATGATGGGWC	95 °C 5 min; 5 cycles of 95 °C 1 min, 58 °C 45 s, 72 °C 2 min; 5 cycles of 95 °C 1 min, 57 °C 45 s, 72 °C 2 min; 35 cycles of 95 °C 1 min, 56 °C 45 s, 72 °C 2 min; 72 °C 10 min; 4 °C soak	O’Donnell et al. (2010)
		F7	Forward	CRACACAGAAGAGTTTGAAGG		
		G2R	Reverse	GTCATYTGDGTDGCDGGYTCDCC		
RNA polymerase second largest subunit	*rpb2*	5f2	Forward	GGGGWGAYCAGAAGAAGGC	95 °C 5 min; 35 cycles of 95 °C 30 s, 55 °C 1 min, 72 °C 1 min; 72 °C 5 min; 4 °C soak	Reeb et al. (2004) Liu et al. (1999)
		7cr	Reverse	CCCATRGCTTGYTTRCCCAT		
partial Beta tubulin	*tub2*	T1	Forward	AACATGCGTGAGATTGTAAGT	95 °C 3 min; 35 cycles of 94 °C 30 s, 54 °C 45 s, 72 °C 15 s; 72 °C 10 min; 4 °C soak	O’Donnell and Cigelnik (1997)
		T2	Reverse	TAGTGACCCTTGGCCCAGTTG		
Calmodulin	*CaM*	CL1	Forward	GARTWCAAGGAGGCCTTCTC	95 °C 1 min; 35 cycles of 94 °C 30 s, 55 °C 30 s, 72 °C 15 s; 72 °C 10 min; 4 °C soak	O’Donnell et al. (2000)
		CL2A	Reverse	TTTTTGCATCATGAGTTGGAC		

^1^ R = A or G; S = C or G; W = A or T; Y = C or T.

**Table 2.** *Fusarium* strains used in this study.

Species name^a^	Isolate^b^	Country/Location	Host/Habitat	GenBank accession numbers^c^				
				*CAM*	*tef1*	*RPB1*	*RPB2*	*tub2*
*Fusariumacaciae*-*mearnsii*	NRRL 25754 T	South Africa	*Acaciamearnsii*	–	AF212448	–	–	AF212765
*F.acuminatum*	LC18312	China, Henan Province, Shangqiu City	Wheat	–	OQ124240	OQ124201	OQ124233	–
*F.acutatum*	NRRL 13308	India	Environmental	–	MN193855	MN193911	MN193883	–
*F.acutatum*	CBS 402.97 T	India	Environmental	MW402459	MW402125	MW402653	MW402768	MW402323
*F.acutatum*	CBS 401.97	India	*Cajanuscajan*	MW402458	MW402124	MW402652	MW402813	MW402322
*F.aethiopicum*	NRRL 46726 T	Ethiopia	*Triticumaestivum*	–	FJ240298	MW233298	MW233470	FJ240288
*F.agapanthi*	NRRL 54463 T	Australia	*Agapanthus* sp.	KU900611	KU900630	KU900620	KU900625	KU900635
*F.agapanthi*	CBS 100193	New Zealand	*Agapanthuspraecox*	MW402363	MW401959	MW402491	MW402727	MW402160
*F.aglaonematis*	ZHKUCC 22-0077 T	China, Guangdong province, Guangzhou city	*Aglaonemamodestum* Schott ex Engl.	ON330434	ON330437	ON330446	ON330443	ON330440
*F.aglaonematis*	ZHKUCC 22-0078	China, Guangdong province, Guangzhou city	*Aglaonemamodestum* Schott ex Engl.	ON330435	ON330438	ON330447	ON330444	ON330441
*F.aglaonematis*	ZHKUCC 22-0079	China, Guangdong province, Guangzhou city	*Aglaonemamodestum* Schott ex Engl.	ON330436	ON330439	ON330448	ON330445	ON330442
*F.algeriense*	NRRL 66647 T	Algeria	*Triticumdurum*	–	MF120510	MF120488	MF120499	–
*F.ananatum*	CBS 118516 T	South Africa	*Ananascomosus* fruit	LT996175	LT996091	LT996188	LT996137	LT996112
*F.andiyazi*	NRRL 31727 T	South Africa	*Sorghumbicolor* soil debris	LT996176	LT996092	LT996189	LT996138	LT996113
*F.andiyazi*	CBS 119856	Ethiopia	Sorghum grain	MN534174	MN533989	MW402523	MN534286	MN534081
*F.anguioides*	NRRL 25385	China	bamboo	–	MH742689	JX171511	JX171624	–
*F.annulatum*	CBS 139739	USA	*Xylosandrusamputatas* galleries in *Cinnamonumcamphora* branch	MW402420	MW402074	MW402602	MW402754	MW402273
*F.annulatum*	CBS 115.97	Italy	*Dianthuscaryophyllus*	MW402373	MW401973	MW402503	MW402785	MW402173
*F.annulatum*	CBS 133.95	Netherlands	*Dianthuscaryophyllus*	MW402407	MW402040	MW402568	MW402743	MW402239
*F.annulatum*	CBS 143605	Iran	Smut	MW402435	MW402094	MW402615	MW402760	MW402293
*F.annulatum*	CBS 792.91	Netherlands	*Gladiolus*	MW402481	MW402153	MW402706	MW402774	MW402354
*F.annulatum*	CBS 137537	Pakistan	*Human tissue*	MW402414	MW402060	MW402586	MW402749	MW402259
*F.annulatum*	NRRL 62905	USA	*Zeamays* kernel	–	MN193865	MW402722	MN193893	–
*F.annulatum*	CBS 258.54 T	New Caledonia	*Oryzasativa*	–	MT010994	MT010944	MT010983	–
*F.annulatum*	LC18417	China, HeBei Province, Xingtai City	Maize	–	OQ126003	OQ125817	OQ126448	OQ126274
*F.annulatum*	LC1105	China	*Lithocarpusglabra*	MW566339	MW580512	MW024500	MW474458	MW533791
*F.annulatum*	LC11490	China, Beijing	*Vitis* sp.	MW566340	MW580513	MW024501	MW474459	MW533792
*F.annulatum*	LC11527	China, Hebei Province	*Vitis* sp.	MW566341	MW580514	MW024502	MW474460	MW533793
*F.annulatum*	LC11584	China, Hebei Province	*Vitis* sp.	MW566342	MW580515	MW024503	MW474461	MW533794
*F.annulatum*	LC11650	China, Hainan Province	*Oryza* sp.	MW566343	MW580516	MW024504	MW474462	MW533795
*F.annulatum*	LC11670	China, Hainan Province	*Oryza* sp.	MW566344	MW580517	MW024505	MW474463	MW533796
*F.annulatum*	LC11672	China, Hainan Province	*Oryza* sp.	MW566345	MW580518	MW024506	MW474464	MW533797
*F.annulatum*	LC13658	China, Neimenggu Province	unidentified mushroom	MW566346	MW580519	MW024507	MW474465	MW533798
*F.annulatum*	LC13659	USA	*Glycinemax*	MW566347	MW580520	MW024508	MW474466	MW533799
*F.annulatum*	LC13660	Philippines	*Musa* sp.	MW566348	MW580521	MW024509	MW474467	MW533800
*F.annulatum*	LC13661	Italy	*Malusdomestica*	MW566349	MW580522	MW024510	MW474468	MW533801
*F.annulatum*	LC13662	Spain	*Chamaeropshumilis*	MW566350	MW580523	MW024511	MW474469	MW533802
*F.annulatum*	LC13663	Ukraine	*Zeamays*	MW566351	MW580524	MW024512	MW474470	MW533803
*F.annulatum*	LC13664	USA	*Sorghumbicolor*	MW566352	MW580525	MW024513	MW474471	MW533804
*F.annulatum*	LC13665	Spain	*Oleaeuropaea*	MW566353	MW580526	MW024514	MW474472	MW533805
*F.annulatum*	LC13666	China, Guangdong Province, Guangzhou city	*Musanana*	MW566354	MW580527	MW024515	MW474473	MW533806
*F.annulatum*	LC13667	China, Guangdong Province, Guangzhou city	*Musanana*	MW566355	MW580528	MW024516	MW474474	MW533807
*F.annulatum*	LC13668	China, Guangdong Province, Guangzhou city	*Musanana*	MW566356	MW580529	MW024517	MW474475	MW533808
*F.annulatum*	LC13669	China, Guangxi Zhuang Autonomous Region, Baise city	*Musanana*	MW566357	MW580530	MW024518	MW474476	MW533809
*F.annulatum*	LC13670	China, Guangxi Zhuang Autonomous Region, Chongzuo city	*Musanana*	MW566358	MW580531	MW024519	MW474477	MW533810
*F.annulatum*	LC13671	China, Guangxi Zhuang Autonomous Region, Laibin city	*Musanana*	MW566359	MW580532	MW024520	MW474478	MW533811
*F.annulatum*	LC13673	China, Hebei Province	*Oryza* sp.	MW566361	MW580534	MW024522	MW474480	MW533813
*F.annulatum*	LC13674	China, Jiangxi Province	*Oryza* sp.	MW566362	MW580535	MW024523	MW474481	MW533814
*F.annulatum*	LC13675	China, Jiangxi Province	*Oryza* sp.	MW566363	MW580536	MW024524	MW474482	MW533815
*F.annulatum*	LC2825	China, Beijing	unidentified grass	MW566364	MW580537	MW024525	MW474483	MW533816
*F.annulatum*	LC5984	China	submerged wood	MW566365	MW580538	MW024526	MW474484	MW533817
*F.annulatum*	LC6002	China	submerged wood	MW566366	MW580539	MW024527	MW474485	MW533818
*F.annulatum*	LC7208	China, Guangdong Province, Guangzhou city	bamboo	MW566367	MW580540	MW024528	MW474486	MW533819
*F.annulatum*	LC7924	China, Shandong Province	*Capsicum* sp.	MW566368	MW580541	MW024529	MW474487	MW533820
*F.anthophilum*	CBS 222.76 ET	Germany	*Euphorbiapulcherrima*	MW402451	MW402114	MW402641	MW402811	MW402312
*F.anthophilum*	NRRL 13602	Germany	*Hippeastrum* sp.	LT996177	LT996093	LT996190	LT996139	LT996114
*F.aquaticum*	LC13615	China, Guizhou Province, Zunyi city	water	–	MW580446	MW024437	MW474392	MW533728
*F.aquaticum*	LC13616	China, Guizhou Province, Zunyi city	water	–	MW580447	MW024438	MW474393	MW533729
*F.aquaticum*	LC7502 T	China, Guizhou Province, Zunyi city	water	–	MW580448	MW024439	MW474394	MW533730
*F.armeniacum*	NRRL 29133 T	Australia	*Triticumaestivum*	–	GQ915501	KT597715	GQ915485	GQ915435
*F.armeniacum*	LC15881	China, Jiangsu Province, Lianyungang City	Maize	–	OQ124873	OQ124463	OQ124668	–
*F.asiaticum*	NRRL 13818 T	Japan	*Hordeumvulgare*	–	AF212451	JX171459	JX171573	AF212768
*F.asiaticum*	LC18286	China, Zhejiang Province, Jiaxing City	Wheat	–	OQ124900	OQ124545	OQ124718	–
*F.atrovinosum*	CBS 445.67 T	Australia	*Triticumaestivum*	MN120693	MN120752	MN120713	MW928822	–
*F.austroafricanum*	NRRL 66741 T	South Africa	Endophyte of *Pennisetumclandestinum*	–	MH742687	MH742537	MH742616	–
*F.avenaceum*	NRRL 26911 NT	Denmark	*Hordeumvulgare*	–	MW928836	MG282372	MG282401	–
*F.avenaceum*	LC18558	China, Gansu Province, Longnan City	Wheat	–	OQ124249	OQ124211	OQ124222	–
*F.awaxy*	LGMF 1930 T	Brazil	Rotten stalks of *Zeamays*	MK766940	MG839004	–	MK766941	MG839013
*F.awaxy*	LC18783	China, Gansu Province, Qingyang City	Maize	OQ125655	OQ126101	OQ125981	OQ126652	OQ126328
*F.aywerte*	NRRL 25410 T	Australia	Soil	–	–	JX171513	JX171626	KU171777
*F.babinda*	NRRL 25807 T	Australia	Soil	MN534162	MN534060	–	MN534245	MN534100
*F.bactridioides*	CBS 100057 T	USA	*Pinusleiophylla*	MN534173	KC514053	MT010939	MT010963	MN534112
*F.bactridioides*	NRRL 20476	USA	*Cronartiumconigenum*	AF158343	AF160290	–	–	U34434
*F.begonia*	NRRL 25300 T	Germany	*Begoniaelatior* hybrid	AF158346	AF160293	LT996191	LT996140	U61543
*F.beomiforme*	NRRL 13606 T	Australia	Soil	–	MF120507	MF120485	MF120496	–
*F.beomiforme*	NRRL 25174	New Caledonia	Soil	–	–	JX171506	JX171619	–
*F.boothii*	NRRL 26916 T	South Africa	*Zeamays*	–	GQ915503	KM361641	KM361659	GQ915437
*F.boothii*	LC18723	China, Gansu Province, Qingyang City	Maize	–	OQ124953	OQ124486	OQ124824	–
*F.brachiariae*	CML 3032 T	Brazil	*Brachiariadecumbens*	–	MT901348	–	MT901314	MT901321
*F.brachiariae*	CML 3163	Brazil	*Brachiariadecumbens*	–	MT901349	–	MT901315	MT901322
*F.brachygibbosum*	NRRL 20954 T	India	*Sorghumvulgare*	–	MW233075	MW233246	MW233418	–
*F.brachygibbosum*	HN-1	China	Maize	–	KX984345	KX984349	KX984353	–
*F.brasilicum*	NRRL 31281 T	Brazil	*Avenasativa*	–	AY452964	–	–	AY452956
*F.brevicatenulatum*	CBS 404.97 T	Madagascar	*Strigaasiatica*	MW834108	MN533995	–	MN534295	MN534063
*F.buharicum*	NRRL 13371	Iran	*Hibiscuscannabinus*	–	OM160859	JX171449	JX171563	–
*F.buharicum*	NRRL 25488 ET	Uzbekistan	*Gossypiumherbaceum*	–	KX302912	KX302920	KX302928	–
*F.bulbicola*	NRRL 13618 T	Germany	*Nerinebowdenii*	KF466327	KF466415	KF466394	KF466404	KF466437
*F.burgessii*	NRRL 66654 T	Australia	Soil	–	HQ667148	MT409440	HQ646393	–
*F.caapi*	CML 3657 T	Brazil	*Brachiariadecumbens*	–	MT901350	–	MT901316	MT901323
*F.caapi*	CML 3658	Brazil	*Brachiariadecumbens*	–	MT901351	–	MT901317	MT901324
*F.caatingaense*	URM 6779 T	Brazil	*Dactylopiusopuntiae*	–	LS398466	–	LS398495	–
*F.caatingaense*	CBS 976.97	USA	*Juniper chinensis*	MN170315	MN170449	–	MN170382	–
*F.camptoceras*	CBS 193.65 NT	Costa Rica	*Theobromacacao*	MN170316	MN170450	MW928800	MN170383	AB820714
*F.casha*	PPRI 21883 T	South Africa	Lesions in *Amaranthuscruentus* associated with *Athesapeutadodonis* and *Barisamaranti* weevils	–	MF787261	–	MN605065	MF787255
*F.casha*	PPRI 20462	South Africa	*Athesapeutadodonis*	–	MF787262	–	MN605066	MF787256
***F.castaneophilum***	**CFCC 70814 T**	**China, BeiJing**	***Castaneamollissima***	**PP946917**	**PP946923**	**PP946937**	**PP946935**	**PP946929**
***F.castaneophilum***	**CFCC 70815**	**China, BeiJing**	***Castaneamollissima***	**PP946918**	**PP946924**	**PP946938**	**PP946936**	**PP946930**
*F.cerealis*	FRC R-4758	USA	Soil	–	MZ921929	MZ921689	MZ921800	–
*F.chinhoyiense*	NRRL 25221 T	Zimbabwe	*Zeamays*	MN534196	MN534050	MW402711	MN534262	MN534082
*F.chinhoyiense*	NY 001B5	South Africa	Soil	MN534197	MN534051	MW402725	MN534263	MN534083
*F.chuoi*	CPC 39664 T	Vietnam	*Musaitinerans*	OK626304	OK626308	OK626306	OK626302	OK626310
*F.chuoi*	CPC 39667	Vietnam	*Musaitinerans*	OK626305	OK626309	OK626307	OK626303	OK626311
*F.circinatum*	CBS 405.97 T	USA	*Pinusradiata*	KM231393	KM231943	JX171510	HM068354	KM232080
*F.clavus*	CBS 126202 T	Namibia	Desert soil	MN170322	MN170456	–	MN170389	–
*F.clavus*	LC18293	China, Hubei Province, Xiangyang City	Wheat	OQ125253	OQ125112	–	OQ125519	–
*F.coffeatum*	CBS 635.76 T	South Africa	*Cynodonlemfuensis*	MN120696	MN120755	MN120717	MN120736	–
*F.coicis*	RBG 5368 T	Australia	*Coixgasteenii*	LT996178	KP083251	KP083269	KP083274	LT996115
*F.commune*	CBS 110090 T	Denmark	Soil	–	AF362263	MW928803	MW934368	–
*F.commune*	LC18583	China, Hunan Province, Hengyang City	Maize	–	OQ125097	OQ125091	OQ125103	–
*F.concentricum*	NRRL 25181 T	Costa Rica	*Musasapientum*	AF158335	AF160282	LT996192	–	U61548
*F.concentricum*	LC18523	China, Guangdong Province, Qingyuan City	Maize	OQ125644	OQ126090	OQ125868	OQ126523	OQ126285
*F.concentricum*	LC1003	China, Guangdong Province, Guangzhou city	*Reineckiacarnea*	–	MW580449	MW024440	MW474395	MW533731
*F.concentricum*	LC11489	China, Beijing	*Vitis* sp.	–	MW580450	MW024441	MW474396	MW533732
*F.concentricum*	LC11491	China, Beijing	*Vitis* sp.	–	MW580451	MW024442	MW474397	MW533733
*F.concentricum*	LC11507	China, Beijing	*Vitis* sp.	–	MW580452	MW024443	MW474398	MW533734
*F.concentricum*	LC13617	China, Jiangsu Province, Changshu city	unknown plant	–	MW580453	MW024444	MW474399	MW533735
*F.concentricum*	LC13618	Japan	*Podocarpusmacrophyllus*	–	MW580454	MW024445	MW474400	MW533736
*F.concentricum*	LC13619	China, Fujian Province, Wuyi Mountain	*Musanana*	–	MW580455	MW024446	MZ399207	MW533737
*F.concentricum*	LC13620	China, Fujian Province, Wuyi Mountain	*Musanana*	–	MW580456	MW024447	MW474402	MW533738
*F.concentricum*	LC13647	China, Fujian Province, Fuzhou city	*Lablab* sp.	MW566324	MW580497	MW024485	MW474443	MW533776
*F.concentricum*	LC13648	China, Fujian Province, Fuzhou city	*Lablab* sp.	MW566325	MW580498	MW024486	MW474444	MW533777
*F.concentricum*	LC13649	China, Fujian Province, Fuzhou city	*Lablab* sp.	MW566326	MW580499	MW024487	MW474445	MW533778
*F.concentricum*	LC4326	China, Jiangxi Province	*Aglaonemamodestum*	MW566288	MW580461	MW024452	MW474407	MW533743
*F.concentricum*	LC4359	China, Jiangxi Province	*Hederanepalensis*	MW566289	MW580462	MW024453	MW474408	MW533744
*F.concentricum*	LC7032	China, Hainan Province	*Musanana*	MW566290	MW580463	MW024454	MW474409	MW533745
*F.concolor*	NRRL 13459	South Africa	Plant debris in soil	GQ505585	GQ505674	JX171455	GQ505852	–
*F.concolor*	NRRL 13994 T	Uruguay	*Hordeumvulgare*	–	MH742650	MH742492	MH742569	–
*F.continuum*	NRRL 66286 T	China	*Zanthoxylumbungeanum*	–	KM236722	KM520387	KM236782	–
*F.cortaderiae*	NRRL 29297 T	New Zealand	*Cortaderiaselloana*	–	AY225885	KM361644	KM361662	AH012625
*F.cugenangense*	NRRL 25387	New Zealand	Human toe nail	–	MH485011	JX171512	JX171625	–
*F.cugenangense*	LC18297	China, Fujian Province, Zhangzhou City	Maize	–	OQ125083	–	OQ125080	–
*F.culmorum*	NRRL 25475 ET	Denmark	*Hordeumvulgare*	–	MW233082	JX171515	JX171628	AF212780
*F.curculicola*	PPRI 20458 T	South Africa	*Athesapeutadodonis*	–	MF787266	–	MN605069	MF787258
*F.curculicola*	PPRI 20386	South Africa	Isolated from lesion in *Amaranthuscruentus* associated with *Athesapeutadodonis* weevils	–	MF787268	–	MN605071	MF787260
*F.curculicola*	PPRI 20464	South Africa	*Athesapeutadodonis*	–	MF787267	–	MN605070	MF787259
*F.nirenbergiae*	CBS 744.97	USA	*Pseudotsugamenziesii*	AF158365	AF160312	LT996203	LT575065	U34424
*F.curvatum*	CBS 238.94 T	Netherlands	*Beaucarnea* sp.	MH484711	MH484984	MW928804	MH484893	MH485075
*F.denticulatum*	NRRL 25302	USA	*Ipomoeabatatas*	AF158322	AF160269	LT996195	LT996143	U61550
*F.denticulatum*	CBS 407.97 T	USA	*Ipomoeabatatas*	MT010890	KR909385	MT010953	MT010970	MT011060
*F.dhileepanii*	BRIP 71717 T	Australia	*Cyperusaromaticus*	–	OK509072	–	OK533536	–
*F.dlaminii*	NRRL 13164 T	South Africa	*Soil debris in cornfield*	AF158330	AF160277	KU171681	KU171701	U34430
*F.dlaminii*	CBS 175.88	South Africa	*Zeamays* soil	MN534150	MN534002	MW402623	MN534256	MN534138
*F.dlaminii*	CBS 671.94	South Africa	Soil	MN534152	MN534004	MW402690	MN534254	MN534136
*F.duoseptatum*	InaCC F916 T	Indonesia	Pseudostem of *Musa* var. Pisang Kepok	–	LS479688	LS479495	LS479239	–
*F.echinatum*	CBS 146496	South Africa	Unidentified tree	MW834109	MW834272	MW834186	MW834003	MW834300
*F.echinatum*	CBS 146497 T	South Africa	Unidentified tree	MW834110	MW834273	MW834187	MW834004	MW834301
*F.elaeagni*	LC18815	China, Fujian Province, Fuzhou City	Rice	OQ125618	OQ126068	OQ125848	OQ126520	OQ126241
*F.elaeagni*	LC13627 T	China, Jiangsu Province, Suzhou city	*Elaeagnuspungens*	MW566293	MW580466	MW024457	MW474412	MW533748
*F.elaeagni*	LC13628	China, Jiangsu Province, Suzhou city	*Elaeagnuspungens*	MW566294	MW580467	MW024458	MW474413	MW533749
*F.elaeagni*	LC13629	China, Jiangsu Province, Suzhou city	*Elaeagnuspungens*	MW566295	MW580468	MW024459	MW474414	MW533750
*F.elaeidis*	CBS 217.49 T	Zaire	*Elaeis* sp.	MH484688	MH484961	MW928805	MH484870	MH485052
*F.equiseti*	NRRL 20697	Chile	*Beta vulgaris*	GQ505506	–	JX171481	JX171595	–
*F.equiseti*	CBS 307.94 ET	Germany	Soil	GQ505511	GQ505599	–	GQ505777	–
*F.erosum*	LC15877 T	China, Guangdong Province, Meizhou City	Maize	OQ125648	OQ126066	OQ125772	OQ126518	OQ126321
*F.fabacearum*	CBS 144743 T	South Africa	*Glycinemax*	MH484757	MH485029	MW928806	MH484938	MH485121
*F.falsibabinda*	LC13611	Japan	*Camelliasasanqua*	MW566261	MW580434	–	MW474380	MW533720
*F.fecundum*	LC15875 T	China, Shaanxi Province, Hanzhong City	Wheat	OQ125281	OQ125250	–	OQ125544	–
*F.ficicrescens*	CBS 125178 T	South Africa	Unidentified tree	KU603958	KU604452	MW402546	KT154002	MT011061
*F.ficicrescens*	CBS 125177	South Africa	Unidentified tree	MN534176	MN534006	MW402545	MN534281	MN534071
*F.flagelliforme*	NRRL 36269 T	Croatia	*Pinusnigra*	GQ505557	GQ505645	–	GQ505823	–
*F.foetens*	CBS 110286 T	Netherlands	*Begoniaelatior* hybrid	–	AY320087	MW928808	MW928825	–
*F.fracticaudum*	CMW 25245 T	Colombia	*Pinusmaximonoii*	–	KJ541059	–	–	KJ541051
*F.fractiflexum*	NRRL 28852 T	Japan	*Cymbidium* sp.	AF158341	AF160288	LR792578	LT575064	–
*F.fredkrugeri*	CBS 144209 T	South Africa	*Melhaniaacuminata* rhizosphere	LT996181	LT996097	LT996199	LT996147	LT996117
*F.fujikuroi*	CBS 221.76 T	Taiwan	*Oryzasativa*	–	–	MW834188	MW834005	–
*F.fujikuroi*	LC18819	China, Sichuan Province, Mianyang City	Maize	OQ125638	OQ126071	OQ125853	OQ126528	OQ126292
*F.fujikuroi*	LC13633	USA	*Glycinemax*	MW566299	MW580472	MW024460	MW474418	MW533751
*F.fujikuroi*	LC13634	Japan	*Acerpalmatum*	MW566300	MW580473	MW024461	MW474419	MW533752
*F.fujikuroi*	LC13635	USA	*Sorghumbicolor*	MW566301	MW580474	MW024462	MW474420	MW533753
*F.fujikuroi*	LC13636	Japan	*Rhododendronsimsii*	MW566302	MW580475	MW024463	MW474421	MW533754
*F.fujikuroi*	LC13637	China, Fujian Province, Wuyi mountain	*Musanana*	MW566303	MW580476	MW024464	MW474422	MW533755
*F.fujikuroi*	LC13638	China, Guangdong Province, Qingyuan city	*Musanana*	MW566304	MW580477	MW024465	MW474423	MW533756
*F.fujikuroi*	LC13639	China, Guangxi Zhuang Autonomous Region, Baise city	*Musanana*	MW566305	MW580478	MW024466	MW474424	MW533757
*F.fujikuroi*	LC13640	China, Guangxi Zhuang Autonomous Region, Liuzhou city	*Musanana*	MW566306	MW580479	MW024467	MW474425	MW533758
*F.fujikuroi*	LC13641	China, Hebei Province	*Oryza* sp.	MW566307	MW580480	MW024468	MW474426	MW533759
*F.fujikuroi*	LC13642	China, Hainan Province, Wanning city	*Panicum* sp.	MW566308	MW580481	MW024469	MW474427	MW533760
*F.fujikuroi*	LC13643	China, Hainan Province, Wanning city	*Panicum* sp.	MW566309	MW580482	MW024470	MW474428	MW533761
*F.fujikuroi*	LC5916	China, Jiangxi Province, Nanchang city	submerged wood	MW566310	MW580483	MW024471	MW474429	MW533762
*F.fujikuroi*	LC5927	China, Jiangxi Province, Nanchang city	submerged wood	MW566311	MW580484	MW024472	MW474430	MW533763
*F.fujikuroi*	LC5945	China, Jiangxi Province, Nanchang city	submerged wood	MW566312	MW580485	MW024473	MW474431	MW533764
*F.fujikuroi*	LC5955	China, Jiangxi Province, Nanchang city	submerged wood	MW566313	MW580486	MW024474	MW474432	MW533765
*F.fujikuroi*	LC5979	China, Jiangxi Province, Nanchang city	submerged wood	MW566314	MW580487	MW024475	MW474433	MW533766
*F.fujikuroi*	LC6014	China, Jiangxi Province, Nanchang city	submerged wood	MW566315	MW580488	MW024476	MW474434	MW533767
*F.fujikuroi*	LC6015	China, Jiangxi Province, Nanchang city	submerged wood	MW566316	MW580489	MW024477	MW474435	MW533768
*F.fujikuroi*	LC6024	China, Jiangxi Province, Nanchang city	submerged wood	MW566317	MW580490	MW024478	MW474436	MW533769
*F.fujikuroi*	LC6973	China, Jiangxi Province	*Citrusreticulata*	MW566318	MW580491	MW024479	MW474437	MW533770
*F.fujikuroi*	LC7147	China, Jiangxi Province	bamboo	MW566319	MW580492	MW024480	MW474438	MW533771
*F.fujikuroi*	LC7864	China, Guangxi Zhuang Autonomous Region	Poaceae sp.	MW566320	MW580493	MW024481	MW474439	MW533772
*F.fujikuroi*	CBS 186.56	Japan	*Oryzasativa* seedling	MW402447	MW402108	MW402632	MW402765	MW402306
*F.fujikuroi*	CBS 257.52	Japan	*Oryzasativa* seedling	MW402454	MW402119	MW402645	MW402812	MW402317
*F.fujikuroi*	CBS 265.54	Unknown	*Oryzasativa*	MN534222	MN534011	MW402650	MN534268	MN534132
*F.fujikuroi*	CBS 119855	Unknown	Environmenta	MW402387	MW401994	–	MW402735	MW402194
*F.fujikuroi*	NRRL 13566	China	*Oryzasativa* culm	AF158332	AF160279	–	JX171570	U34415
*F.gaditjirrii*	NRRL 45417	Unknown	Unknown	–	MN193881	MN193937	MN193909	–
*F.gaditjirrii*	NRRL 53678 T	Australia	*Heteropogontriticeus*	AY639631	AY639636	–	HQ662690	AY639626
*F.gerlachii*	NRRL 36905 T	USA	*Triticumaestivum*	–	DQ459742	KM361646	KM361664	–
*F.gigantean*	1-F T	Brazil	*Panicummaximum*	–	OR610357	–	OR578833	–
*F.globosum*	NRRL 26131 T	South Africa	*Zeamays*	KF466329	KF466417	KF466396	KF466406	KF466439
*F.globosum*	CBS 430.97	South Africa	*Zeamays*	MN534219	MN534013	–	MN534265	MN534125
*F.globosum*	CBS 120992	South Africa	Maize kernels	MW402390	MW401998	MW402529	MW402788	MW402198
*F.globosum*	CBS 431.97	South Africa	*Zeamays*	MW402465	MW402131	MW402669	MW402816	MW402330
*F.glycines*	CBS 144746 T	South Africa	*Glycinemax*	MH484760	MH485033	MW928809	MH484942	MH485124
*F.goolgardi*	RBG 5411 T	Australia	*Xanthorrhoeaglauca*	–	KP101123	KP083270	KP083280	–
*F.gossypinum*	CBS 116613 T	Ivory Coast	*Gossypiumhirsutum*	MH484727	MH485000	–	MH484909	MH485091
*F.graminearum*	LC18796	China, Shandong Province, Dezhou City	Maize	–	OQ124986	OQ124494	OQ124787	–
*F.graminearum*	NRRL 31084	USA	*Zeamays*	–	MW233103	JX171531	MW233447	HQ141668
*F.graminum*	NRRL 20692	Unknown	*Paspalum*, *Vicia*	–	–	JX171479	JX171593	–
*F.guilinense*	LC12160 T	China	*Musanana*	MK289652	MK289594	MK289831	MK289747	MW533851
*F.guttiforme*	CBS 409.97 T	Brazil	*Ananascomosus*	MT010901	KC514066	MT010938	MT010967	MT011048
*F.hainanense*	LC11638 T	China	*Oryza* sp.	MK289657	MK289581	MK289833	MK289735	MW533852
*F.hainanense*	LC18701	China, Guangxi Zhuang Autonomous Region, Guigang City	Maize	OQ125282	OQ125148	–	OQ125490	–
*F.hechiense*	LC13644 T	China, Guangxi Zhuang Autonomous Region,Hechi city	*Musanana*	MW566321	MW580494	MW024482	MW474440	MW533773
*F.hechiense*	LC13645	China, Guangxi Zhuang Autonomous Region,Hechi city	*Musanana*	MW566322	MW580495	MW024483	MW474441	MW533774
*F.hechiense*	LC13646	China, Guangxi Zhuang Autonomous Region,Hechi city	*Musanana*	MW566323	MW580496	MW024484	MW474442	MW533775
*F.heterosporum*	NRRL 20693	Netherlands	*Clavicepspurpurea* on *Loliumperenne*	–	–	JX171480	JX171594	–
*F.heterosporum*	CBS 391.68 ET	Germany	*Clavicepspurpurea* on *Loliumperenne*	–	MW928839	MW928811	MW928827	–
*F.hoodiae*	CBS 132474 T	South Africa	Root of *Hoodiagordonii*	MH484747	MH485020	–	MH484929	MH485111
*F.hostae*	NRRL 29889 T	USA	*Hosta* sp.	–	AY329034	JX171527	JX171640	AY329042
*F.humuli*	CQ1039 T	China	*Humulusscandens*	MK289712	MK289570	MK289840	MK289724	MW533857
*F.incarnatum*	NRRL 25478 ET	Malawi	*Tricho*-*santhes dioica*	MN170342	MN170476	–	MN170409	–
*F.incarnatum*	ITEM 7155	Unknown	Unknown	LN901597	LN901581	–	LN901617	LN901630
*F.ipomoeae*	LC18759	China, Shandong Province, Jinan City	Maize	OQ125260	OQ125122	–	OQ125529	–
*F.jinanense*	LC15878 T	China, Shandong Province, Jinan City	Maize	OQ125271	OQ125131	–	OQ125521	–
*F.konzum*	CBS 119849 T	USA	*Sorghastrumnuttans*	LT996182	LT996098	LT996200	LT996148	LT996118
*F.kyushuense*	NRRL 3509 T	Japan	*Triticumaestivum*	–	MH582292	MW233227	MH582098	–
*F.kyushuense*	LC18277	China, Sichuan Province, Suining City	Wheat	–	OQ125072	OQ124662	OQ124671	–
*F.lactis*	NRRL 25200 ET	USA	*Ficuscarica*	AF158325	AF160272	LT996201	LT996149	U61551
*F.lactis*	CBS 420.97	USA	*Ficuscarica*	MN534181	MN534015	MW402667	MN534296	MN534078
*F.langsethiae*	CBS 113234 T	Norway	*Avenasativa*	–	AB674298	MW928812	MW928828	AB587069
*F.languescens*	CBS 645.78 T	Morocco	*Solanumlycopersicum*	MH484698	MH484971	MW928813	MH484880	MH485062
*F.lateritium*	NRRL 13622	USA	*Ulmus* sp.	–	–	JX171457	JX171571	–
*F.libertatis*	CBS 144749 T	South Africa	Rock surface	MH484762	MH485035	–	MH484944	MH485126
*F.longicornicola*	NRRL 52706 T	Ethiopia	Insect	MW402487	JF740788	–	–	MW402360
*F.longicornicola*	NRRL 52712	Ethiopia	Insect	MW402488	JF740794	MW402716	–	MW402361
*F.longipes*	NRRL 20723	England	Unknown	–	–	JX171483	JX171596	–
*F.longipes*	NRRL 20695 T	USA	Soil	–	GQ915509	MW233244	GQ915493	GQ915443
*F.louisianense*	NRRL 54197 T	USA	Seeds of *Triticum* sp	–	KM889633	KM889655	KM889657	KM889628
*F.luffae*	LC12167 T	China	*Luffaaegyptiaca*	MK289698	MK289601	MK289869	MK289754	–
*F.mangiferae*	Indo63	Indonesia	*Musa* sp. var. Pisang Raja Nangka	–	LS479441	–	LS479850	LS479433
*F.lumajangense*	LC13650	China, Guangxi Zhuang Autonomous Region Chongzuo city	*Musanana*	MW566328	MW580501	MW024489	MW474447	MW533780
*F.lumajangense*	LC13651	China, Guangxi Zhuang Autonomous Region Chongzuo city	*Musanana*	MW566329	MW580502	MW024490	MW474448	MW533781
*F.lumajangense*	LC13652	China, Guangxi Zhuang Autonomous Region	*Arengacaudata*	MW566330	MW580503	MW024491	MW474449	MW533782
*F.lyarnte*	NRRL 54252 T	Australia	Soil	–	EF107118	JX171549	JX171661	–
*F.madaense*	LC13614	China, Hebei Province	*Oryza* sp.	MW566272	MW580445	MW024436	MW474391	MW533727
*F.madaense*	CBS 146656	Nigeria	*Arachishypogaea*	MW402438	MW402097	MW402618	MW402763	MW402296
*F.madaense*	CBS 146669 T	Nigeria	*Arachishypogaea*	MW402439	MW402098	MW402619	MW402764	MW402297
*F.mangiferae*	NRRL 53980 T	Israel	*Mangiferaindica*	–	LT574978	MW402530	LT575059	MN534128
*F.mangiferae*	NRRL 25226	Israel	*Mangiferaindica*	AF158334	AF160281	JX171509	HM068353	U61561
*F.marasasianum*	CMW 25512	Colombia	*Pinustecunumanii*	MN534208	MN534018	–	MN534249	MN534113
*F.meridionale*	NRRL 28436 T	New Caledonia	*Citrussinensis*	–	AF212435	KM361642	KM361660	AF212752
*F.meridionale*	LC18774	China, Yunnan Province, Xuanwei City	Maize	–	OQ125048	OQ124527	OQ124830	–
*F.mesoamericanum*	NRRL 25797 T	Honduras	*Musa* sp.	–	AF212441	KM361639	KM361657	AF212758
*F.mexicanum*	NRRL 53147 T	Mexico	*Mangiferaindica*	–	MG838032	MG838088	MN724973	MG838107
*F.mexicanum*	NRRL 47473	Mexico	*Mangiferaindica*	GU737389	GU737416	LR792579	LR792615	GU737308
*F.mianyangense*	LC15879 T	China, Sichuan Province, Mianyang City	Rice	OQ125335	OQ125232	–	OQ125510	–
*F.mirum*	CML 3859 T	Mallawi, Egypt	*Sorghumbicolor*	–	MK895725	–	MK907308	MK907329
*F.mirum*	CML 3858	Mallawi, Egypt	*Sorghumbicolor*	–	MK895726	–	MK907307	MK907328
*F.mirum*	KSU 15077	Bokle, Cameroon	*Sorghumbicolor*	–	MT374735	–	MT374738	MT374740
*F.mirum*	LLC929	Ethiopia	Soil	OP485896	OP487012	–	OP486581	–
*F.miscanthi*	NRRL 26231 T	Denmark	*Miscanthussinensis*	–	MN193878	JX171521	JX171634	KU171785
*F.mundagurra*	RBG5717 T	Australia	Soil	–	KP083256	KP083272	KP083276	MT901328
*F.mundagurra*	LC13689	China, Hainan Province	*Paspalumvaginatum*	MW566383	MW580556	MW024544	MW474502	MW533835
*F.mundagurra*	LGS129.2	China, Hainan Province	*Paspalumvaginatum*	MZ399201	MZ399211	MZ399204	MZ399208	MZ399214
*F.mundagurra*	LGS129.3	China, Hainan Province	*Paspalumvaginatum*	MZ399202	MZ399212	MZ399205	MZ399209	MZ399215
*F.musae*	NRRL 25059 T	Honduras	*Musa* sp.	FN552064	FN552086	MW402689	FN552108	FN545368
*F.musae*	NRRL 28893	Mexico	*Musa* sp.	FN552070	FN552092	–	FN552114	FN545374
*F.nanum*	LC12168 T	China	*Musanana*	MK289651	MK289602	MK289871	MK289755	–
*F.napiforme*	NRRL 13604 T	Namibia	*Pennisetumtyphoides*	AF158319	AF160266	HM347136	EF470117	U34428
*F.napiforme*	CBS 135139	India	Keratitis (Human)	MN534183	MN534019	MW402572	MN534290	MN534084
*F.nelsonii*	CBS 119876 T	South Africa	*Triticum* soil	GQ505374	GQ505404	MN120722	GQ505468	–
*F.nelsonii*	NRRL 13338	Australia	Soil	GQ505372	MW233225	MW233397	MW233569	–
*F.nepalense*	NRRL 54222 T	Nepal	*Oryzasativa*	–	KM889631	KM361650	KM361668	–
*F.newnesense*	NRRL 66241 T	Australia	Soil	–	KP083261	–	–	–
*F.nirenbergiae*	CBS 744.97	Unknown	Unknown	AF158365	AF160312	–	LT575065	U34424
*F.nirenbergiae*	CBS 840.88 T	Netherlands	*Dianthuscaryophyllus*	MH484705	MH484978	–	MH484887	MH485069
*F.nisikadoi*	NRRL 25308 T	Japan	*Triticumaestivum*	–	KR909358	MG282391	MG282421	–
*F.nodosum*	CBS 201.63 T	Portugal	*Arachishypogaea*	MN120704	MN120763	MN120725	MN120743	–
*F.nodosum*	NRRL 36351	Lisboa, Portugal	*Storedpeanuts*	–	MW233117	MW233289	MW233461	–
*F.nothincarnatum*	LC18436 T	China, Heilongjiang Province, Daqing City	Rice	–	OQ125147	–	OQ125509	–
*F.nurragi*	NRRL 36452	Australia	Soil	–	–	JX171538	JX171650	–
*F.nurragi*	CBS 393.96 T	Australia	Soil	–	MW928840	MW928814	MW928830	–
*F.nygamai*	NRRL 13448 T	Australia	*Sorghumbicolor*	AF158326	AF160273	LT996202	EF470114	U34426
*F.nygamai*	CBS 413.97	Morocco	Oryzasativa	MW402462	MW402127	MW402660	MW402815	MW402325
*F.odoratissimum*	Indo8 T	Indonesia	*Musa* sp. cv. Pisang Kepok	–	LS479828	LS479618	LS479386	–
*F.ophioides*	CBS 118512 T	South Africa	*Panicummaximum*	MN534209	EU921239	–	MN534303	MN534118
*F.ophioides*	CBS 118515	South Africa	*Panicummaximum*	MN534205	MN534025	–	MN534298	MN534120
*F.oxysporum*	CBS 144134	Germany	*Solanumtuberosum*	MH484771	MH485044	–	MH484953	MH485135
*F.palustre*	NRRL 54056 T	USA	*Spartinaalterniflora*	–	MW233131	MW233303	KT597731	MH875687
*F.palustre*	NRRL 54050	USA	*Spartinaalterniflora*	–	–	KT597717	KT597729	–
*F.panlongense*	LC13656 T	China, Guangxi Zhuang Autonomous Region,Guilin city	*Musanana*	MW566337	MW580510	MW024498	MW474456	MW533789
*F.parvisorum*	CMW 25267	Colombia	*Pinuspatula*	–	KJ541060	–	–	KJ541055
*F.pharetrum*	CBS 144751 T	South Africa	*Aloidendrondichotomum*	MH484770	MH485043	MW928815	MH484952	MH485134
*F.phyllophilum*	NRRL 13617 T	Italy	*Dracaenaderemensis*	KF466333	KF466421	KF466399	KF466410	KF466443
*F.pilosicola*	NRRL 29124 T	USA	*Bidenspilosa*	MN534159	MN534055	–	MN534248	MN534099
*F.pilosicola*	NRRL 29123	USA	*Bidenspilosa*	MN534165	MN534054	–	MN534247	MN534098
*F.pininemorale*	CMW 25243 T	Colombia	*Pinustecunumanii*	MN534211	MN534026	–	MN534250	MN534115
*F.planum*	LC15876 T	China, Guangdong Province, Qingyuan City	Maize	OQ125677	OQ126125	OQ125871	OQ126555	OQ126352
*F.poae*	NRRL 13714	Canada	Overwintered wheat	–	–	JX171458	JX171572	–
*F.poae*	NRRL 26941 ET	USA	infected barley kernel	–	–	KU171686	KU171706	KU171786
*F.poae*	LC18712	China, Qinghai Province, Haidong City	Maize	–	OQ125075	OQ124666	OQ124674	–
*F.praegraminearum*	NRRL 39664 T	New Zealand	Litter in maize paddock	–	KX260120	KX260125	KX260126	KX260131
*F.prieskaense*	CBS 146498 T	South Africa	*Prunusspinosa*	MW834112	MW834275	MW834190	MW834007	MW834303
*F.prieskaense*	CBS 146499	South Africa	*Prunusspinosa*	MW834113	MW834276	MW834191	MW834008	MW834304
*F.proliferatum*	F026	China, Zhejiang Province, Ningbo city	*Musa* sp.	MZ399203	MZ399213	MZ399206	MZ399210	MZ399216
*F.proliferatum*	CBS 480.96 ET	Papua New	Tropical rain forest soil	MN534217	MN534059	–	MN534272	MN534129
*F.pseudoanthophilum*	CBS 414.97 T	Zimbabwe	*Zeamays*	MW402463	MT011006	MT010949	MT010980	MW402326
*F.pseudoanthophilum*	CBS 415.97	Zimbabwe	*Zeamays*	–	MW402129	MW402662	–	MW402327
*F.pseudoanthophilum*	CBS 745.97	Zimbabwe	*Zeamays*	MW402476	MW402148	MW402697	MW402820	MW402349
*F.pseudocircinatum*	NRRL 22946 T	Ghana	*Solanum* sp.	AF158324	AF160271	LT996204	LT996151	U34427
*F.pseudocircinatum*	CBS 455.97	Papua New	*Heteropsyllaincisa*	MN534184	MN534029	–	MN534276	MN534070
*F.pseudocircinatum*	LC13676	China, Taiwan Province	*Syzygiumsamarangense*	MW566369	MW580542	MW024530	MW474488	MW533821
*F.pseudocircinatum*	LC13677	China, Taiwan Province	*Syzygiumsamarangense*	MW566370	MW580543	MW024531	MW474489	MW533822
*F.pseudograminearum*	NRRL 28062 T	Australia	*Hordeumvulgare*	–	AF212468	JX171524	JX171637	AF107867
*F.pseudonygamai*	NRRL 13592 T	Nigeria	*Pennisetumtyphoides*	AF158316	AF160263	LT996205	LT996152	U34421
*F.pseudonygamai*	CBS 416.97	Nigeria	*Pennisetumtyphoides*	MN534194	MN534030	MW402663	MN534283	MN534064
*F.ramigenum*	NRRL 25208 T	USA	*Ficuscarica*	KF466335	MN193867	MN193923	MN193895	KF466445
*F.ramigenum*	CBS 526.97	USA	*Ficuscarica*	MN534188	MN534032	MW402682	MN534292	MN534086
*F.redolens*	NRRL 22901	Canada	*Pseudotsugamenziesii*	–	MT409452	JX171503	JX171616	–
*F.redolens*	NRRL 25600 ET	Germany	*Pisumsativum*	–	MT409453	MT409433	MT409443	AY329040
***F.rubicola***	**CFCC 70816 T**	**China, Shaanxi Province**	***Rubuslambertianus***	**PP946919**	**PP946925**	**PP946939**	–	**PP946931**
***F.rubicola***	**CFCC 70817**	**China, Shaanxi Province**	***Rubuslambertianus***	**PP946920**	**PP946926**	**PP946940**	–	**PP946932**
***F.rubicola***	**CFCC 70818**	**China, Shaanxi Province**	***Rubuslambertianus***	**PP946921**	**PP946927**	**PP946941**	–	**PP946933**
***F.rubicola***	**CFCC 70819**	**China, Shaanxi Province**	***Rubuslambertianus***	**PP946922**	**PP946928**	**PP946942**	–	**PP946934**
*F.sacchari*	NRRL 13999 ET	India	*Saccharumofficinarum*	AF158331	AF160278	JX171466	JX171580	U34414
*F.sacchari*	LC1058	China, Guangdong Province, Guangzhou city	*Arundinagraminifolia*	MW566371	MW580544	MW024532	MW474490	MW533823
*F.sacchari*	LC13625	Philippines	*Musa* sp.	MW566291	MW580464	MW024455	MW474410	MW533746
*F.sacchari*	LC13626	China, Guangdong Province, Guangzhou city	*Musanana*	MW566292	MW580465	MW024456	MW474411	MW533747
*F.sacchari*	LC13657	China, Guangxi Zhuang Autonomous Region, Baise city	*Musanana*	MW566338	MW580511	MW024499	MW474457	MW533790
*F.sacchari*	LC13678	China, Guangdong Province, Guangzhou city	*Musanana*	MW566372	MW580545	MW024533	MW474491	MW533824
*F.sacchari*	LC13679	China, Guangxi Zhuang Autonomous Region, Qinzhou city	*Musanana*	MW566373	MW580546	MW024534	MW474492	MW533825
*F.sacchari*	LC13680	China, Guangxi Zhuang Autonomous Region, Qinzhou city	*Musanana*	MW566374	MW580547	MW024535	MW474493	MW533826
*F.sacchari*	LC13681	China, Beijing	*Poaannua*	MW566375	MW580548	MW024536	MW474494	MW533827
*F.sambucinum*	NRRL 22187	England	*Solanumtuberosum*	–	MW834277	JX171493	JX171606	KM232078
*F.sanyaense*	LC15882 T	China, Hainan Province, Sanya City	Maize	OQ125641	OQ126093	OQ125859	OQ126547	OQ126322
*F.sarcochroum*	NRRL 20472 NT	Switzerland	*Viscumalbum*	–	MW834278	JX171472	JX171586	–
*F.scirpi*	NRRL 13402	Australia	Soil	GQ505504	GQ505592	JX171452	JX171566	–
*F.scirpi*	NRRL 36478 ET	Australia	Soil	GQ505566	GQ505654	–	GQ505832	–
*F.secorum*	NRRL 62593 T	USA	*Beta vulgaris*	KJ189235	KJ189225	–	–	–
*F.secorum*	NRRL 62594	USA	*Beta vulgaris*	KJ189238	KJ189228	–	–	–
*F.sibiricum*	NRRL 53430 T	Russia	*Avenasativa*	–	HM744684	MW233302	HQ154472	HQ141659
*F.siculi*	CPC 27188 T	Italy	*Citrussinensis*	–	LT746214	LT746299	LT746327	–
*F.siculi*	CPC 27189	Italy	*Citrussinensis*	LT746190	LT746215	–	LT746328	LT746347
*F.sororula*	CMW 40578 T	Colombia	*Pinuspatula*	LT996184	KJ541067	LT996206	LT996153	KJ541057
*F.spartum*	NRRL 66896 T	Tunisia	Rhizosphere of *Macrochloatenacissima*	–	MT409459	MT409439	MT409449	–
*F.sporotrichioides*	NRRL 3299 T	France	Corn	–	DQ676612	JX171444	DQ676587	HQ141641
*F.sterilihyphosum*	NRRL 25623 T	South Africa	*Mangiferaindica*	–	MN193869	MW402713	MN193897	–
*F.stilboides*	NRRL 20429	Nyasaland	*Coffea* sp.	–	–	JX171468	JX171582	–
*F.stilboides*	CBS 746.79 ET	Cook Islands	*Citrus* sp.	–	MW928843	MW928817	MW928832	–
*F.subglutinans*	NRRL 22016 NT	USA	*Zeamays*	AF158342	AF160289	JX171486	JX171599	U34417
*F.subglutinans*	LC18249	China, Heilongjiang Province, Yichun City	Maize	OQ125674	OQ126108	OQ125971	OQ126671	OQ126342
*F.subglutinans*	CBS 215.76	Germany	*Zeamays*	MN534171	MN534061	MW402636	MN534241	MN534109
*F.subglutinans*	CBS 479.94	South Africa	*Zeamays* kernel	MN534166	MN534036	MW402678	MN534236	MN534105
*F.subglutinans*	LC13682	USA	*Glycinemax*	MW566376	MW580549	MW024537	MW474495	MW533828
*F.subglutinans*	LC13683	USA	*Zeamays*	MW566377	MW580550	MW024538	MW474496	MW533829
*F.subglutinans*	LC13684	Canada	*Glycinemax*	MW566378	MW580551	MW024539	MW474497	MW533830
*F.subglutinans*	LC13685	Canada	*Glycinemax*	MW566379	MW580552	MW024540	MW474498	MW533831
*F.subglutinans*	LC13686	Canada	*Glycinemax*	MW566380	MW580553	MW024541	MW474499	MW533832
*F.sublunatum*	NRRL 13384	Costa Rica	Soil	KM231389	–	JX171451	JX171565	KM232076
*F.subtropicale*	NRRL 66764 T	Brazil	*Hordeumvulgare*	–	MH706974	MH706972	MH706973	MH706968
*F.succisae*	NRRL 13613 ET	Germany	*Succisapratensis*	AF158344	AF160291	LT996207	LT996154	U34419
*F.sudanense*	CBS 454.97 T	Sudan	*Strigahermonthica*	LT996185	KU711697	LT996208	LT996155	KU603909
*F.sudanense*	CBS 675.94	Sudan	*Strigahermonthica*	MN534182	MN534038	MW402693	MN534279	MN534074
*F.sulawesiense*	InaCC F940 T	Indonesia	*Musaacuminata* var.	–	LS479443	–	LS479855	–
*F.sulawesiense*	LC18688	China, Jiangsu Province, Lianyungang City	Maize	OQ125344	OQ125216	–	OQ125467	–
*F.sulawesiense*	LC13723	China, Guangxi Zhuang Autonomous Region	*Smilaxcorbularia*	MW574215	MW594393	–	MW474536	MW533906
*F.tanahbumbuense*	InaCC F965 T	Indonesia	*Musa* var. Pisang Hawa	LS479432	LS479448	LS479877	LS479863	–
*F.tanahbumbuense*	CBS 145.44	Unknown	Unknown	MN170371	MN170505	–	MN170438	–
*F.tanahbumbuense*	LC18534	China, Hainan Province, Lingao County	Maize	OQ125392	OQ125140	–	OQ125499	–
*F.temperatum*	MUCL 52463 T	Belgium	*Zeamays*	MW402486	KM487197	–	MW402776	MW402359
*F.temperatum*	LC18813	China, Yunnan Province, Xuanwei City	Maize	OQ125672	OQ126118	OQ125989	OQ126670	OQ126332
*F.temperatum*	NRRL 25622	South Africa	*Zeamays*	AF158354	AF160301	–	–	AF160317
*F.temperatum*	LC5848	China, Guizhou Province	unidentified lichen	MW566327	MW580500	MW024488	MW474446	MW533779
*F.terricola*	CBS 483.94 T	Australia	Soil	KU603951	KU711698	LT996209	LT996156	KU603908
*F.terricola*	CBS 119850	Australia	Soil	MN534180	MN534041	MW402520	MN534280	MN534075
*F.thapsinum*	NRRL 22045	South Africa	*Sorghumbicolor*	LT996186	AF160270	JX171487	JX171600	U34418
*F.thapsinum*	CBS 776.96 T	Unknown	Unknown	KU603967	MN534044	MW402704	MN534289	MN534080
*F.thapsinum*	CBS 539.79	Italy	Man, white grained mycetoma	MW402472	MW402140	MW402686	MW402818	MW402340
*F.thapsinum*	LC13687	USA	*Sorghumbicolor*	MW566381	MW580554	MW024542	MW474500	MW533833
*F.thapsinum*	LC13688	USA	*Glycinemax*	MW566382	MW580555	MW024543	MW474501	MW533834
*F.tjaetaba*	RBG 5361 T	Australia	*Sorghuminterjectum*	LT996187	KP083263	MW834192	KP083275	–
*F.torreyae*	NRRL 54149	USA	*Torreya* sp.	–	HM068337	JX171548	JX171660	–
*F.torulosum*	NRRL 22748	Netherlands	Boxwood	–	OL772877	JX171502	JX171615	–
*F.trachichlamydosporum*	CBS 102028	Malaysia	*Musasapientum*	MH484715	MH484988	–	MH484897	MH485079
*F.transvaalense*	CBS 144211 T	South Africa	*Sidacordifolia*	–	LT996099	LT996210	LT996157	–
*F.transvaalense*	NRRL 31008	Australia	Soil	–	MW233102	MW233274	MW233446	–
*F.tricinctum*	NRRL 25481 ET	Germany	Winter wheat culm base	–	AB674263	JX171516	JX171629	–
*F.triseptatum*	CBS 258.50 T	USA	*Ipomoeabatatas*	MH484691	MH484964	MW928820	MH484873	MH485055
*F.tupiense*	NRRL 53984 T	Brazil	*Mangiferaindica*	GU737377	GU737404	LR792583	LR792619	–
*F.udum*	NRRL 22949	Germany	*Lactariuspubescens*	AF158328	AF160275	LT996220	LT996172	U34433
*F.udum*	BBA 65058 ET	India	*Cajanuscajan*	–	MK639096	–	KY498875	KY498892
*F.ussurianum*	NRRL 45681 T	Russia	*Avenasativa*	–	FJ240301	KM361648	KM361666	–
*F.venenatum*	NRRL 22196	Germany	*Zeamays*	–	MW233078	JX171494	JX171607	–
*F.verticillioides*	LC18525	China, Anhui Province, Chuzhou City	Maize	OQ125739	OQ126150	OQ125919	OQ126583	OQ126357
*F.verticillioides*	NRRL 22172	Germany	*Zeamays*	AF158315	AF160262	LT996221	EF470122	U34413
*F.verticillioides*	CBS 218.76 ET	Germany	*Zeamays*	MW402449	KF499582	MW402638	MW928835	MW402311
*F.verticillioides*	LC18464	China, Liaoning Province, Shenyang City	Maize	OQ125698	OQ126216	–	OQ126649	OQ126379
*F.verticillioides*	LC13653	Brazil	*Glycinemax*	MW566331	MW580504	MW024492	MW474450	MW533783
*F.verticillioides*	LC13654	USA	*Glycinemax*	MW566332	MW580505	MW024493	MW474451	MW533784
*F.verticillioides*	LC13655	China, Guangdong Province, Guangzhou city	*Musanana*	MW566333	MW580506	MW024494	MW474452	MW533785
*F.verticillioides*	LC2810	China, Sichuan Province, Zhangjiajie	bamboo	MW566334	MW580507	MW024495	MW474453	MW533786
*F.verticillioides*	LC2818	China, Beijing	*Physosfegiavirginiana*	MW566335	MW580508	MW024496	MW474454	MW533787
*F.verticillioides*	LC5896	China, Jiangxi Province, Nanchang city	submerged wood	MW566336	MW580509	MW024497	MW474455	MW533788
*F.veterinarium*	NRRL 36153 T	Netherlands	Peritoneum of *Selachimorpha*	MH484717	MH484990	–	MH484899	MH485081
*F.volatile*	CBS 143874 T	French Guiana	Human bronchoalveolar lavage fluid	MK984595	LR596007	–	LR596006	LR596008
*F.vorosii*	NRRL 37605 T	Hungary	*Triticumaestivum*	–	DQ459745	KM361647	KM361665	–
*F.weifangense*	LC18333 T	China, Shandong Province, Weifang City	Wheat	OQ125276	OQ125107	–	OQ125515	–
*F.werrikimbe*	CBS 125535 T	Australia	*Sorghumleiocladum*	MN534203	MW928846	MW928821	MN534304	MN534104
*F.xylarioides*	NRRL 25486 ET	Ivory Coast	*Coffea* sp.	MW402455	AY707136	JX171517	HM068355	AY707118
*F.xyrophilum*	NRRL 62721 T	Guyana	*Xyrissurinamensis*	–	MN193877	MN193933	MN193905	–
*F.xyrophilum*	NRRL 62710	Guyana	*Xyrissurinamensis*	–	MN193875	MW402720	MN193903	–
*F.zanthoxyli*	NRRL 66285 T	China	*Zanthoxylumbungeanum*	–	OM160879	OM160837	OM160858	–
*Fusicollaviolacea*	CBS 634.76 T	Iran	*Quadraspidiotusperniciosus*	KM231407	KM231956	KM232251	–	KM232095

a The new species names, described in this study are in bold. b Abbreviations for the culture collections: the U.S. Agricultural Research Service culture collection (NRRL); the Westerdijk Fungal Biodiversity Institute (WI) collection (CBS); the working collection of FABI (CMW) of the Forestry and Agricultural Biotechnology Institute (FABI), University of Pretoria, South Africa; the working collection of the author Neriman Yilmaz (NY), University of Pretoria, South Africa; (BBA) Julius Kühn-Institute, Institute for Epidemiology and Pathogen Diagnostics, Berlin & Braunschweig, Germany; the University Recife Mycology culture collection at the Universidade Federal de Pernambuco, Recife, Brazil (URM); Mycotheque de lUniversite Catholique de Louvain, Louvain-la-Neuve, Belgium (MUCL); Lei Cai’s personal culture collection (LC), the Institute of Microbiology, Chinese Academy of Sciences, Beijing, China; Indonesian culture collection (InaCC); Zhongkai University of Agriculture and Engineering (ZHKUCC); South African National Collection of Fungi (PPRI); (CPC) Collection of P.W. Crous, held at WI; (FRC) Fusarium Research Center, University Park, PA, USA. c The sequences deposited to GenBank in this study are in bold. ET: Ex-epitype, NT: Ex-neotype, HT: Ex-holotype, T: Ex-type.

### ﻿Phylogenetic analyses

Sequences were aligned in MAFFT v. 7 at the web server (http://mafft.cbrc.jp/alignment/server) (Katoh et al. 2019) and further corrected manually using MEGA7.0.21 (Kumar et al. 2016). Phylogenies were calculated for each gene individually, followed by a concatenated dataset of the five genes (each gene region was treated as a separate partition) using Maximum Likelihood (ML) and Bayesian Inference (BI) methods. Using RAxMLHPC Blackbox 8.2.10 (Stamatakis 2014) on the CIPRES Science Gateway portal to perform Maximum Likelihood (ML) analysis (https://www.phylo.org), the GTR-GAMMA model was selected, and a total of 1000 bootstrap replicates were run. The Bayesian posterior probability (BPP) was determined using Markov chain Monte Carlo (MCMC) sampling in MrBayes v.3.2.6 (Ronquist et al. 2012). The six Markov chains run simultaneously for 1 million generations from a random tree, with tree sampling occurring every 100 generations. For accuracy, 25% of the aged samples were discarded and the analysis was continued until the mean standard deviation of the split frequencies was below 0.01. The phylogram was visualized in FigTree v.1.3.1 (http://tree.bio.ed.ac.uk/software) and edited using Adobe Illustrator CS5 (Adobe Systems Inc., USA).

The genealogical concordance phylogenetic species recognition (GCPSR) model was used to analyze phylogenetically related but ambiguous species, and a pairwise homoplasy index (PHI) test was performed. A pairwise homoplasy index (PHI) test (Philippe and Bryant 2006) was conducted in SplitsTree (Huson 1998; Huson and Bryant 2006) to assess the level of recombination among phylogenetically closely related species, using both the LogDet transformation and splits decomposition options. This analysis utilized a concatenated five-locus dataset (*tef1*, *CaM*, *rpb1*, *rpb2*, and *tub2*) from closely related species. If the pairwise homoplasy index results were below a 0.05 threshold (Φw < 0.05), it indicated significant recombination in the dataset. The relationships between closely related species were visualized by constructing splits graph.

### ﻿Morphological observations

*Fusarium* species were characterized and described based on the previously defined macroscopic and microscopic morphological characteristics (Crous et al. 2021). After incubating at 25 °C in darkness for 7 days, approximately 5 × 5 mm agar plates were taken from the edge of colonies on SNA (synthetic nutrient-poor agar; Nirenberg 1976) and transferred to the culture medium for morphological characterization. Colony morphology, production of pigments, and odors were documented on PDA, OA (oatmeal agar; Crous et al. 2019), and SNA after incubation for 7 days at 25 °C in darkness. Colony color codes were determined following the protocols of Kornerup and Wanscher (1978).

For morphological comparison, cultures on CLA (carnation leaf agar; Fisher et al. 1982) should be incubated at 25 °C for 7–14 days under a 12/12-hour near-ultraviolet/dark cycle to observe micromorphological characteristics, including sporodochia, conidiophores, phialides, conidia (both sporodochial and aerial), and chlamydospores. Morphological characteristics were examined and photodocumented using water as a mounting medium under a Leica DM 2500 dissecting microscope (Wetzlar, Germany) and a Nikon Eclipse 80i compound microscope equipped with differential interference contrast (DIC) illumination. Images were captured with a Nikon DS-Ri2 camera and processed using the Nikon NIS Elements F4.30.01 software. For each species, 30 phialides and chlamydospores, and 50 conidia were randomly measured to calculate the mean, standard deviation, and minimum-maximum values. Taxonomic novelties were deposited in MycoBank (http://www.mycobank.org).

## ﻿Results

### ﻿Phylogeny

**Figure 1.**
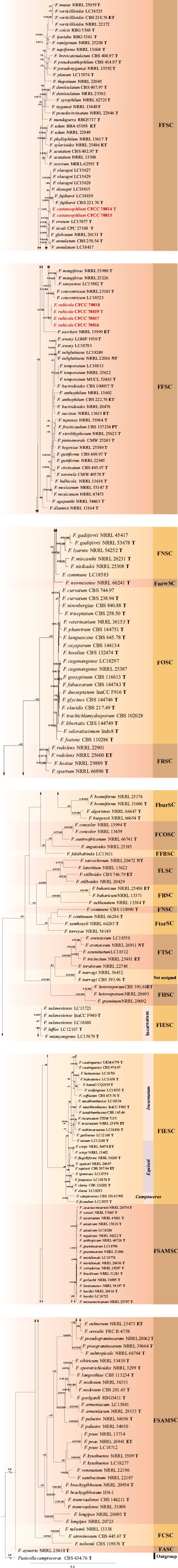
Fifty percent majority rule consensus tree from a Bayesian analysis based on a three-locus combined dataset (*tef1*, *rpb1*, and *rpb2*) illustrating the phylogenetic relationship between six isolates of *Fusarium* and the *Fusarium* species complex. The Bayesian posterior probabilities (PP > 0.95) and PhyML Bootstrap support values (BS > 50) are displayed at the nodes (PP/ML). The tree was rooted to *Fusicollaviolacea* (CBS 634.76 T). Ex-type cultures are indicated with ‘T’, epi-type with ‘ET’, neotype with ‘NT’.

The Bayesian Inference (BI) and Maximum Likelihood (ML) phylogenetic analyses of the phylogeny of the six isolates of *Fusarium* fungi produced topologically similar trees. The analysis was conducted using a combined dataset of *tef1*, *rpb1*, and *rpb2*, which contained 3 241 characters, including gaps. This dataset comprised 777 bp for *tef1*, 1 559 bp for *rpb1*, and 898 bp for *rpb2*. *Fusicollacamptoceras* CBS 634.76 (ex-type) was used as the outgroup taxon. The combined *tef1*, *rpb1*, and *rpb2* phylogeny (Fig. 1) revealed that the six isolates clustered into the *Fusariumfujikuroi* species complex (FFSC).

**Figure 2.**
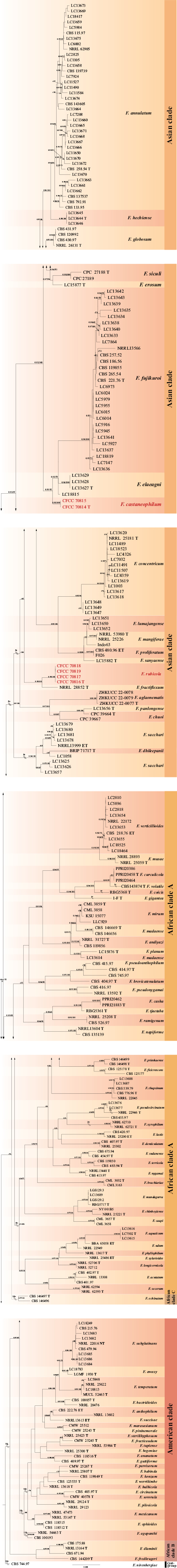
Fifty percent majority rule consensus tree from a Bayesian analysis based on a five-locus combined dataset (*tef1*, *CaM*, *rpb1*, *rpb2*, and *tub2*) showing the phylogenetic relationships of species within the *Fusariumfujikuroi* species complex (FFSC). The Bayesian posterior probabilities (PP > 0.9) and PhyML Bootstrap support values (BS > 50) are displayed at the nodes (PP/ML). The tree was rooted to *F.nirenbergiae* (CBS 744.97). New species are indicated in bold, ex-type cultures with ‘T’, epi-type with ‘ET’, neotype with ‘NT’.

In order to conduct further phylogenetic analysis on 6 strains of *Fusarium*, a multigene phylogeny was used to reveal the identities of the FFSC (Fig. 2). The alignment contained 269 taxa and was 4 342 bp long, including the gaps. This dataset comprised 681 bp for *tef1*, 668 bp for *CaM*, 1 555 bp for *rpb1*, 879 bp for *rpb*2, and 535 bp for *tub2*. In addition, individual gene phylogenies were generated to assess the genealogical concordance of the novel species of FFSC (Suppl. material 1: figs S1–S5). Genealogical concordance analyses subsequently confirmed the distinctiveness of the two novel species described in this study. The two new species are now recognized as *F.castaneophilum* and *F.rubicola*.

**Figure 3.**
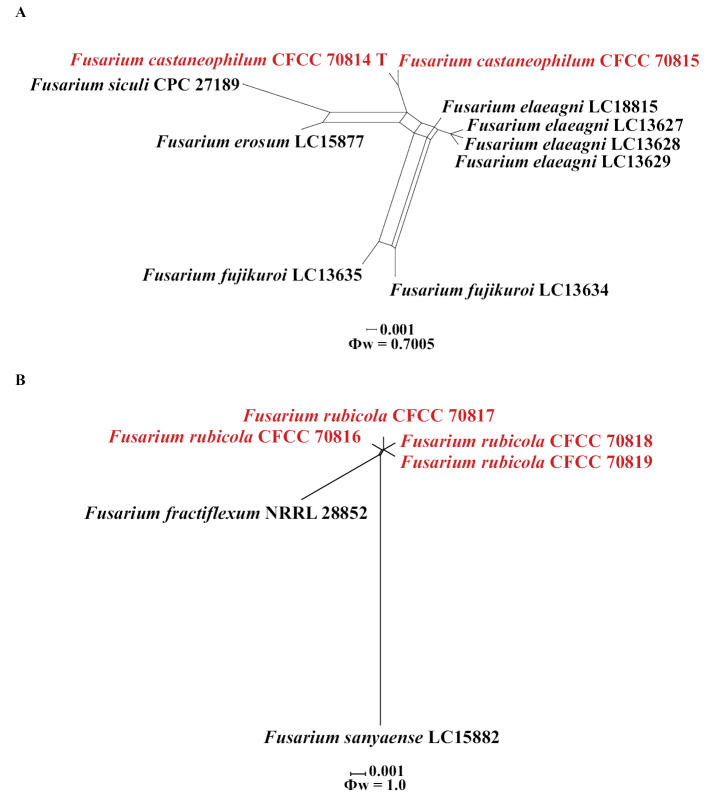
The results of the pairwise homoplasy index (PHI) test for two newly described taxa and closely related species were obtained from the five-locus concatenated datasets (*tef1*, *CaM*, *rpb1*, *rpb2*, and *tub2*), using both LogDet transformation and splits decomposition **A** the PHI of *Fusariumcastaneophilum* sp. nov. with their phylogenetically related isolates or species **B** the PHI of *Fusariumrubicola* sp. nov. with their phylogenetically related isolates or species. PHI test results (Φw) < 0.05 indicate significant recombination within the dataset.

Applying the Genealogical Concordance Phylogenetic Species Recognition (GCPSR) concept to *F.castaneophilum*, we selected *F.elaeagni* (LC18815, LC13627, LC13628, LC13629), *F.erosum* (LC15877), *F.fujikuroi* (LC13634, LC13635) and *F.siculi* (CPC 27189), as closely related species. The concatenated sequence dataset of five-loci (*tef1*, *CaM*, *rpb1*, *rpb2* and *tub2*) underwent the Population History Index (PHI) test, revealing that no significant recombination was observed among these isolates/taxa (Φw = 0.7005) (Fig. 3A). This finding provided strong support for the proposition that these isolates belonged to three distinct taxa.

Applying the Genealogical Concordance Phylogenetic Species Recognition (GCPSR) concept to *F.rubicola*, we selected *F.fractiflexum* (NRRL 28852) and *F.sanyaense* (LC15882) as closely related species. The concatenated sequence dataset of five-loci (*tef1*, *CaM*, *rpb1*, *rpb2* and *tub2*) underwent the Population History Index (PHI) test, revealing that no significant recombination was observed among these isolates/taxa (Φw = 1.0) (Fig. 3B), which strongly supported the proposition that these isolates belonged to three distinct taxa.

### ﻿Taxonomy

In this section, Latin binomials are provided for the two novel phylospecies resolved in this study, namely *F.castaneophilum* and *F.rubicola*.

#### ﻿ Fusarium castaneophilum

Taxon classificationFungiHypocrealesNectriaceae

M.W. Zhang & C.M. Tian sp. nov.

2D38C09E-CD4B-552E-8A5B-C8661081BAD8

 854421

Fig. 4

##### Type.

China • BeiJing, Huairou District *Castanea* Technology Test and Promotion Station (40°25'37.21"N, 116°32'42.83"E), on branch of *Castaneamollissima*, 9 Oct 2022, Y. Ren, holotype BJFC-HR08, ex-type living culture CFCC 70814.

**Figure 4.**
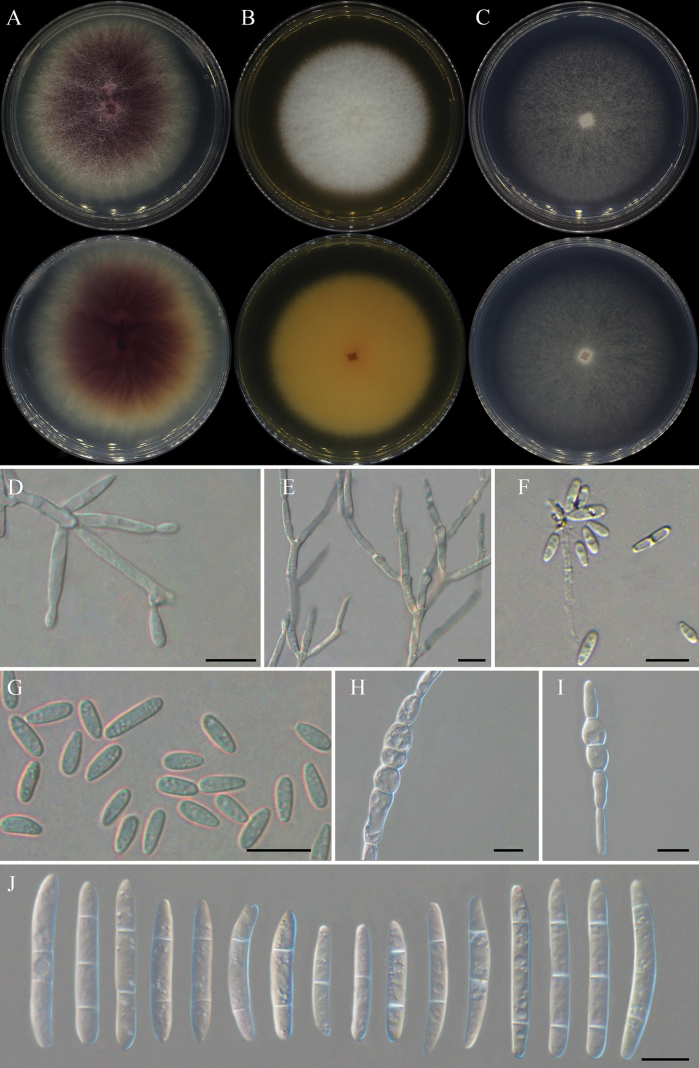
*Fusariumcastaneophilum* (ex-type culture CFCC 70814) **A** colony on PDA**B** colony on OA **C** colony on SNA **D, E** aerial conidiophores and conidiogenous cells **F, G** aerial microconidia **H, I** chlamydospores **J** aerial macroconidia. Scale bars: 10 μm (**D–K**)

##### Etymology.

Named after the host genus from which it was isolated, *Castanea*.

##### Description.

Conidiophores in aerial mycelia, 12–49 μm tall, simple or loosely irregularly branched, bearing terminal or intercalary polyphialides, smooth- and thin-walled, 5.9–35.8 × 1.9–4.3 (av. ± sd. 14.2 ± 6.5 × 2.7 ± 0.5 μm), periclinal thickening inconspicuous or absent; aerial conidia hyaline, smooth- and thin-walled, of two types: (a) microconidia ellipsoidal, obovoid to subclavate, 0–1-septate: 4.5–12 × 2–3.6 μm (av. ± sd. 7.2 ± 1.5 × 2.7 ± 0.3 μm); (b) macroconidia clavate to falcate, straight or dorsiventrally curved, with a blunt to slightly papillate apical cell and a blunt to barely notched or foot-like basal cell, smooth- and thin-walled, 1–3-septate; 1-septate conidia: 14.7–48.6 × 2.3–7.5 μm (av. ± sd. 28.9 ± 8.1 × 4.8 ± 1.3 μm); 2-septate conidia: 24.6–70.4 × 2.2–7.1 μm (av. ± sd. 39.9 ± 8.9 × 5.1 ± 0.9 μm); 3-septate conidia: 26.6–90.4 × 2.2–7.3 μm (av. ± sd. 56.3 ± 14.1 × 5 ± 1.2 μm). Chlamydospores formed in pairs or forming chains, intercalary, globose to subglobose, 7.3–9.5 µm diam, thick-walled, smooth. Sporodochia not observed.

##### Culture characteristics.

Colonies on PDA growing in the dark reaching 7.8–8.0 cm diam after 7 days at 25 °C, optimal 25–30 °C (after 7 days), raised, aerial mycelia dense, colony margin filamentous, surface vinaceous purple in the center, pale luteous at the margin; reverse dark purple in the center, pure yellow at the margin. Colonies on OA growing in the dark reaching 6.8–7 cm diam after 7 days at 25 °C, raised, aerial mycelia dense, colony margin entire, surface white; reverse orange in the center, luteous at the margin. Colonies on SNA grown in the dark reaching 6.4–6.6 cm diam after 7 days at 25 °C, flat, aerial mycelia scant, colony margin entire, white; reverse white. Pigment and odor absent.

##### Notes.

The isolates of *F.castaneophilum* were phylogenetically closely related to *F.elaeagni* (ex-type, LC 13627) isolated from *Elaeagnuspungens* in China (Fig. 2). There were 24 nucleotide position differences between the two species (7/658 in *tef1*, 1/591 in *CaM*, 9/891 in *rpb1*, 3/879 in *rpb2*, 4/490 in *tub2*). The PHI analysis showed that there was no significant recombination between *F.castaneophilum* isolates and its related species (Φw = 0.7005) (Fig. 3A). Morphologically, *F.elaeagni* did not produce any pigment, and the aerial mycelium on PDA was raised, aerial mycelia dense, sporodochia were grayish-orange, and abundantly formed on carnation leaves. However, *F.castaneophilum* produces a purple pigment, and the mycelium on PDA is sparser than the former, no obvious protruding colonies. Microscopically, *F.castaneophilum* has chlamydospores and its aerial phialides are longer than *F.elaeagni*, microconidia are slightly larger than *F.elaeagni*. Thus, *F.castaneophilum* is recognized as a novel species in FFSC.

#### ﻿ Fusarium rubicola

Taxon classificationFungiHypocrealesNectriaceae

M.W. Zhang & C.M. Tian sp. nov.

BFB4BE57-4105-5A42-A0BA-FE0ABA74D807

 854422

Fig. 5

##### Type.

China • Shaanxi Province, Ningshan County Huoditang Shibazhang Waterfall Park (33°23'56.23"N, 108°22'9.93"E), on leaf of *Rubuslambertianus*, 19 Jul 2021, S.J. Li, holotype BJFC-H187, ex-type living culture CFCC 70819.

**Figure 5.**
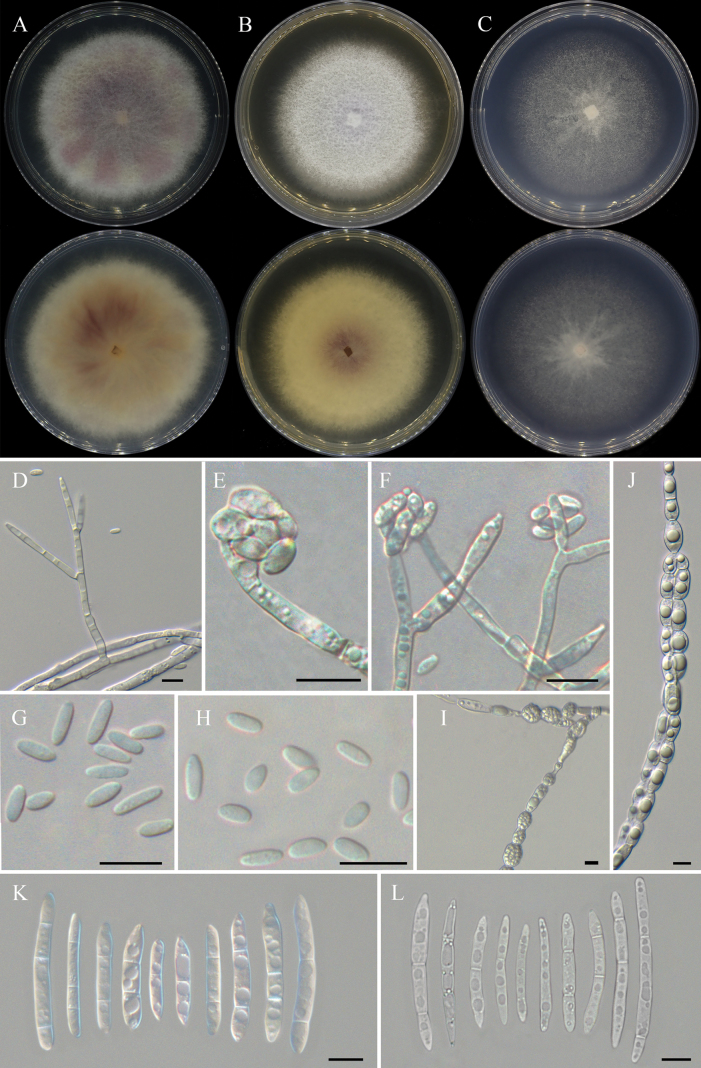
*Fusariumrubicola* (ex-type culture CFCC 70816) **A** colony on PDA**B** colony on OA **C** colony on SNA **D–F** aerial conidiophores, phialides, and aerial conidia **G, H** aerial microconidia **I, J** chlamydospores **K, L** aerial macroconidia. Scale bars: 10 μm (**D–K**).

##### Etymology.

Named after the host genus from which it was isolated, *Rubus*.

##### Description.

Conidiophores in aerial mycelia, 27–90 μm tall, straight or flexuous, smooth- and thin-walled, unbranched, sympodial or irregularly branched, bearing terminal or lateral phialides; aerial phialides polyphialides, subulate to subcylindric, smooth- and thin-walled, periclinal thickening inconspicuous or absent, 5.9–35.8 × 1.9–4.3 μm; aerial conidia hyaline, smooth- and thin-walled, of two types: (a) microconidia ellipsoidal to clavate, aseptate, 7.9–42.7 × 1.7–4.3 μm (av. ± sd. 22.6 ± 7.6 × 3.0 ± 0.6 μm), clustering in discrete false heads at the phialide tips; (b) macroconidia slightly clavate to falcate, straight or gently dorsiventrally curved, with a blunt to slightly papillate apical cell and a blunt to gently notched basal cell, smooth- and thin-walled, 1–3-septate; 1-septate conidia: 15.9–41.5 × 2.7–5.1 μm (av. ± sd. 24.9 ± 4.4 × 4 ± 0.5 μm); 2-septate conidia: 27–51 × 2.9–6.4 μm (av. ± sd. 36.7 ± 5.6 × 4.6 ± 0.8 μm); 3-septate conidia: 26.9–82 × 2.1–6.3 μm (av. ± sd. 53.7 ± 11.7 × 4.6 ± 0.9 μm). Chlamydospores abundant, globose, subglobose to ovoid, subhyaline, smooth-walled, intercalary, solitary, in pairs or forming chains, 7.4–14.9 μm diam. Sporodochia not observed.

##### Culture characteristics.

Colonies on PDA growing in the dark reaching 7.8–8.0 cm diam after 7 days at 25 °C, optimal 25–30 °C (after 7 days), raised, aerial mycelia dense, colony margin entire, surface pale violet to mauve in the center, pale luteous to white at the margin; reverse livid purple in the center, pure yellow at the margin. Colonies on OA growing in the dark reaching 7.0–7.2 cm diam after 7 days at 25 °C, raised, aerial mycelia dense, colony margin crimp, surface pale purple in the center, white at the marginwhite; reverse vinaceous purple in the center, pale luteous at the margin. Colonies on SNA grown in the dark reaching 6.0–6.2 cm diam after 7 days at 25 °C, slightly raised, aerial mycelia scant, colony margin entire, white; reverse white. Pigment and odor absent.

##### Notes.

The isolates of *F.rubicola* were phylogenetically closely related to *F.fractiflexum* (ex-type, NRRL 28852) isolated from *Cymbidium* ssp. in Japan (Fig. 2). There were 15 nucleotide position differences between the two species (8/667 in *tef1*, 2/645 in *CaM*, 5/865 in *rpb1*). The PHI analysis showed that there was no significant recombination between *F.rubicola* isolates and its related species (Φw = 1.0) (Fig. 3B). Morphologically, *F.fractiflexum* forms yellowish colonies, while *F.rubicola* forms purple colonies on the PDA. Microscopically, the microconidia of *F.fractiflexum* are 0–3 septate, forming zigzag-like conidial chains, without chlamydospores. Nevertheless, the microconidia of *F.rubicola* are aseptate, lack zigzag-like conidial chains, and inherence chlamydospores. In conclusion, the phylogenetic and morphological evidence support this fungus being a new species within the FFSC.

## ﻿Discussion

The genus *Fusarium* was first discovered by Link (1809) and established based on *F.roseum*. At that time, the main morphological characteristics of *Fusarium* were determined to be unique canoe-like or banana-like conidia. However, several other genera also produce spores in this form, so it may be incorrect to identify *Fusarium* based solely on morphology. With the development of molecular biology, molecular technology has been widely applied in fungal taxonomy and systematics. The phylogenetic species established based on molecular biology can compensate for some deficiencies of morphological species, reflect the phylogenetic relationship of *Fusarium* more scientifically, and provide a scientific basis and technical methods for rapid molecular detection and identification of strains. The current study reveals that the FFSC comprises more than 60 accepted species, including numerous cryptic species that can only be identified through phylogenetic analysis. In this study, morphology, phylogeny, and Pathogenicity-Related Index (PHI) tests were combined to identify two new species within the *Fusariumfujikuroi* species complex (FFSC). Phylogenetic analyses of a five-gene dataset strongly supported the uniqueness of the two *Fusarium* phylospecies identified, with robust monophyletic statistical support values.

Research indicates that the FFSC (*Fusariumfujikuroi* species complex) comprises three primary clades, which were classified at the time as the Asian, American, and African clades. (O’Donnell et al. 1998a). Subsequently, they found that the African branch was divided into three sub-branches: African Clade A, African Clade B, and African Clade C. In this study, we confirmed that the African clade is not monophyletic, and that *F.dlaminii* and *F.fredkrugeri* form a separate group (the African clade B). This is consistent with the studies of Herron et al. (2015), Sandoval-Denis et al. (2018b), and Wang et al. (2022). And the study found that the African clade C forms a sister clade of the American clade and African clade B, which is consistent with the findings of Crous et al. (2021). This experiment further verified and clarified that the African clade is not monophyletic. Two of the newly named species in this study, *F.castaneophilum* and *F.rubicola*, were resolved in the Asian clade.

*Fusarium* is a notorious plant pathogen that can lead to the death of the host, decrease the yield of cash crops, and result in significant economic losses. This study isolated two new species from *Castaneamollissima* and *Rubuslambertianus*. *Fusariumcastaneophilum* was isolated from chestnut plants (*C.mollissima*). *Castaneamollissima* is an important economic tree species widely distributed in Asia, America, Africa, and Europe (Sun et al. 2022). Wang et al. (2006) reported that a significant number of *Fusarium* species have been isolated from the *C.mollissima* fruit rot disease in Beijing, but the specific species have not been identified. Numerous studies have shown that *Fusarium* poses a threat to the safety of *C.mollissima* in China. In which, *F.proliferatum* (FFSC), *F.graminearum* (FSAMSC), *F.equiseti* (FIESC), and *F.solani* (*Neocosmospora* currently) have all been reported as pathogens of *C.mollissima* in Guizhou, Beijing, Hebei, and other locations in China (Zhang et al. 2022; Zhu 2024). *Fusariumcastaneophylum* may be a potential pathogenic fungus for *C.mollissima*, and its pathogenicity requires further study.

In this study, *F.rubicola* was isolated from *Rubuslambertianus*. There is currently limited research on *Fusarium* affecting *R.lambertianus*. Studies have found that *Fusarium* can make other plants in *Rubus* (such as *R.idaeus*, *R.fruticosus*, etc.) susceptible to diseases (Pastrana et al. 2021; Yang et al. 2024). Among them, Zhang et al. (2013) found that the pathogen responsible for *R.idaeus* stem blight is *F.equiseti* (FIESC), while the pathogen causing *R.idaeus* fruit rot is *F.avenaceum* (FTSC). In addition, Heilongjiang and Yunnan provinces have also discovered fungi from the *Fusarium* that damage plants of *Rubus*. *Fusariumrubicola* may be a potential pathogenic fungus for *R.lambertianus*, requiring further research on its pathogenicity.

The newly described *Fusarium* species in this study have not been linked to any pathogenic effects on their hosts. However, they should not be ignored, as the host range of several species in the FFSC has not yet been determined. For some researchers, it may be irrelevant to describe species without information regarding its pathogenic and/or mycotoxigenic potential. Regardless, it is still of utmost importance to better understand the biodiversity of a specific *Fusarium* species. This study provides Latin binomials for two new species, which will facilitate the opportunity to more easily identify other isolates of these species in future research.

## Supplementary Material

XML Treatment for Fusarium castaneophilum

XML Treatment for Fusarium rubicola

## ﻿Additional information

### Author ORCIDs

Mingwei Zhang https://orcid.org/0009-0006-9906-1234

Cheng Peng https://orcid.org/0009-0005-9619-8246

Shuji Li https://orcid.org/0009-0006-4734-8399

Chengming Tian https://orcid.org/0000-0002-3352-7664

### Data availability

All of the data that support the findings of this study are available in the main text or Supplementary Information.

## Supplementary materials

Supplementary material 1 Phylogeny of the different genes region of species from the *Fusariumfujikuroi* species complex Data type zip Explanation note **fig. S1.** Phylogeny of the *tef1* gene region of species from the *Fusariumfujikuroi* species complex. *F.nirenbergiae* (CBS 744.97) was selected as the out-group. Strains belonging to new species are indicated in bold. Bootstrap values (≥50%) are indicated above branches. Ex-type cultures are indicated with ‘T’, epi-type with ‘ET’, and neotype with ‘NT’. **fig. S2.** Phylogeny of the *CaM* gene region of species from the *Fusariumfujikuroi* species complex. *F.nirenbergiae* (CBS 744.97) was selected as the out-group. Strains belonging to new species are indicated in bold. Bootstrap values (≥50%) are indicated above branches. Ex-type cultures are indicated with ‘T’, epi-type with ‘ET’, and neotype with ‘NT’. **fig. S3.** Phylogeny of the *rpb1* gene region of species from the *Fusariumfujikuroi* species complex. *F.nirenbergiae* (GUCC 197140.2) was selected as the out-group. Strains belonging to new species are indicated in bold. Bootstrap values (≥50%) are indicated above branches. Ex-type cultures are indicated with ‘T’, epi-type with ‘ET’, and neotype with ‘NT’. **fig. S4.** Phylogeny of the *rpb2* gene region of species from the *Fusariumfujikuroi* species complex. *F.nirenbergiae* (CBS 744.97) was selected as the out-group. Strains belonging to new species are indicated in bold. Bootstrap values (≥50%) are indicated above branches. Ex-type cultures are indicated with ‘T’, epi-type with ‘ET’, and neotype with ‘NT’. **fig. S5.** Phylogeny of the *tub2* gene region of species from the *Fusariumfujikuroi* species complex. *F.nirenbergiae* (CBS 744.97) was selected as the out-group. Strains belonging to new species are indicated in bold. Bootstrap values (≥50%) are indicated above branches. Ex-type cultures are indicated with ‘T’, epi-type with ‘ET’, and neotype with ‘NT’. https://binary.pensoft.net/file/1221163This dataset is made available under the Open Database License (http://opendatacommons.org/licenses/odbl/1.0/). The Open Database License (ODbL) is a license agreement intended to allow users to freely share, modify, and use this Dataset while maintaining this same freedom for others, provided that the original source and author(s) are credited.Mingwei Zhang, Cheng Peng, Shuji Li, Chengming Tian
