# Supplementary figures and images for: ﻿Morphological and phylogenetic analyses reveal two new species of the Fusariumfujikuroi (Hypocreales, Nectriaceae) species complex in China
Source: MycoKeys. 2025 Jan 16;112:127–63. doi: 10.3897/mycokeys.112.133472 (PMC11758097; doi:10.3897/mycokeys.112.133472)

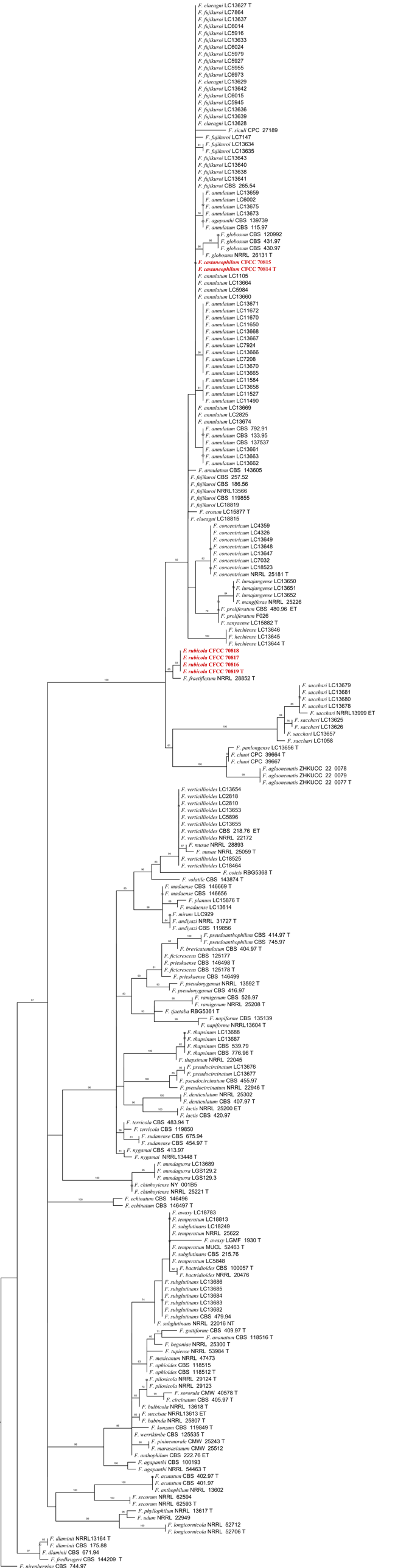

Supplement: Supplementary material 1 — Phylogeny of the different genes region of species from the Fusariumfujikuroi species complex [file mycokeys-112-127-s001.zip › Figure S2.pdf]

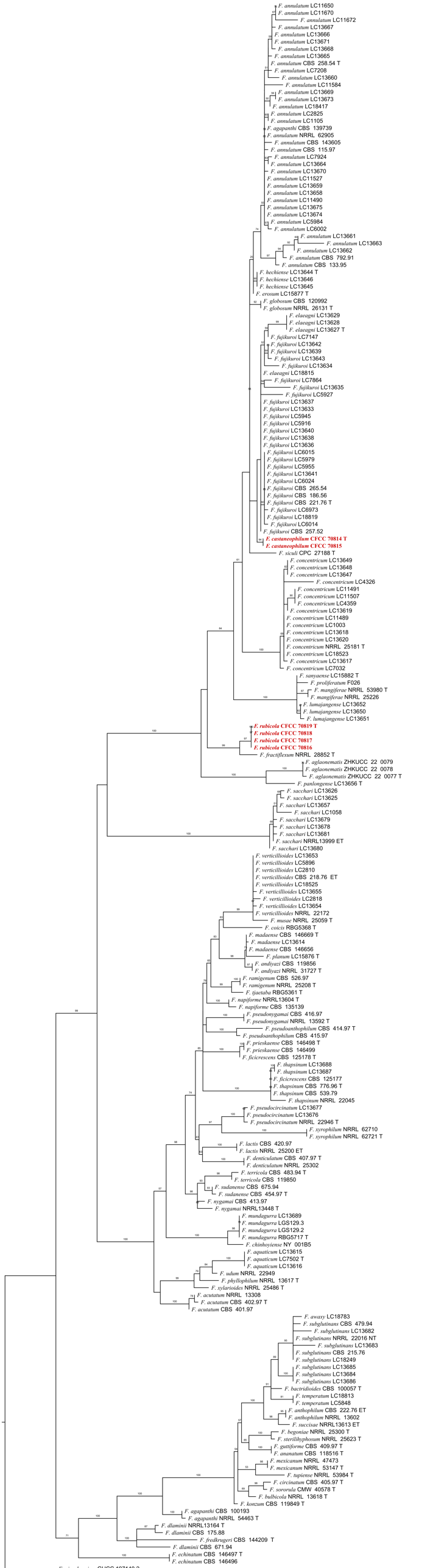

Supplement: Supplementary material 1 — Phylogeny of the different genes region of species from the Fusariumfujikuroi species complex [file mycokeys-112-127-s001.zip › Figure S3.pdf]

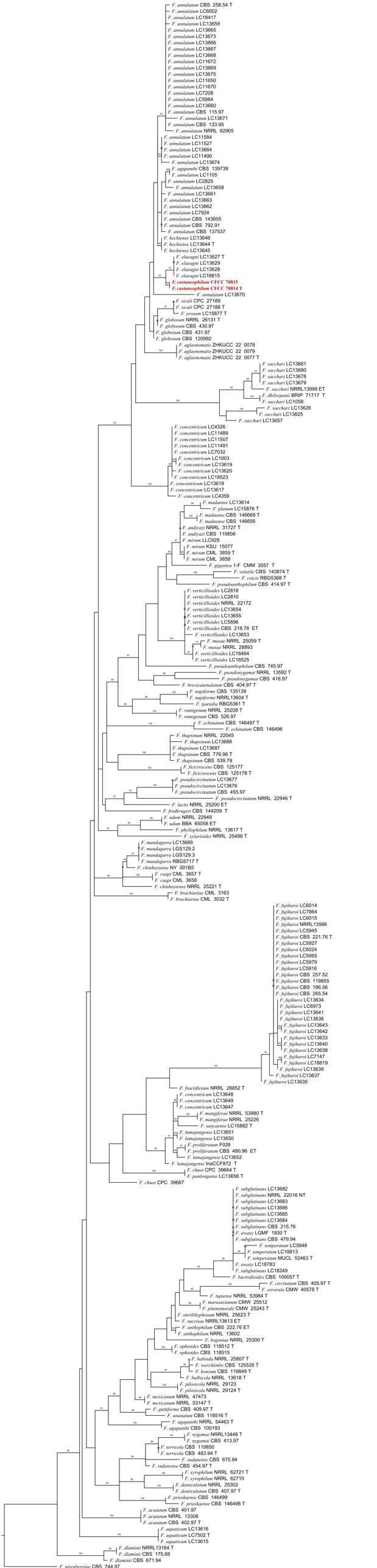

Supplement: Supplementary material 1 — Phylogeny of the different genes region of species from the Fusariumfujikuroi species complex [file mycokeys-112-127-s001.zip › Figure S4.pdf]

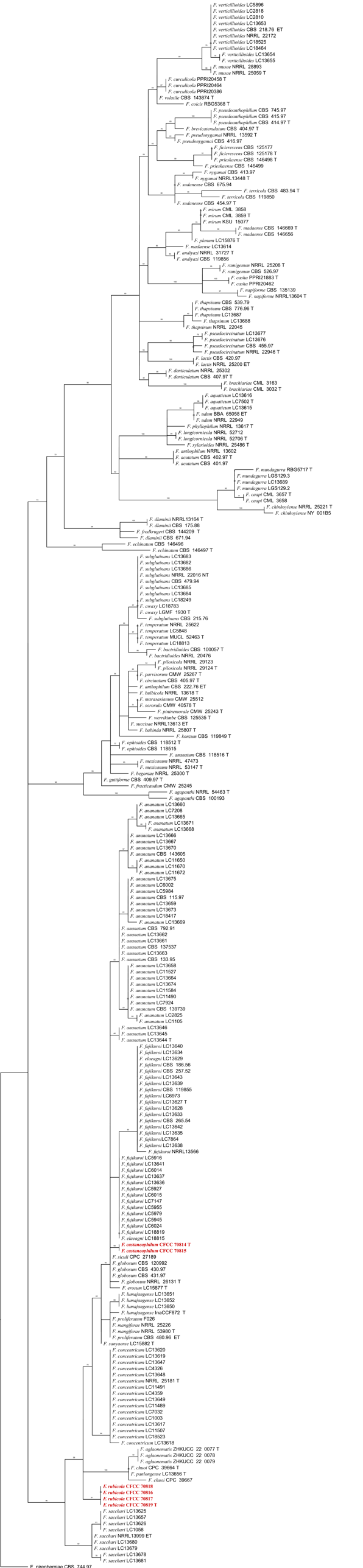

Supplement: Supplementary material 1 — Phylogeny of the different genes region of species from the Fusariumfujikuroi species complex [file mycokeys-112-127-s001.zip › Figure S5.pdf]
