# Supplementary material for: ﻿Morphological and phylogenetic analyses reveal two new species of the Fusariumfujikuroi (Hypocreales, Nectriaceae) species complex in China
Source: MycoKeys. 2025 Jan 16;112:127–63. doi: 10.3897/mycokeys.112.133472 (PMC11758097; doi:10.3897/mycokeys.112.133472)
Supplement: Supplementary material 1 — Phylogeny of the different genes region of species from the Fusariumfujikuroi species complex [file Figure S1.pdf › Figure S1.pdf]

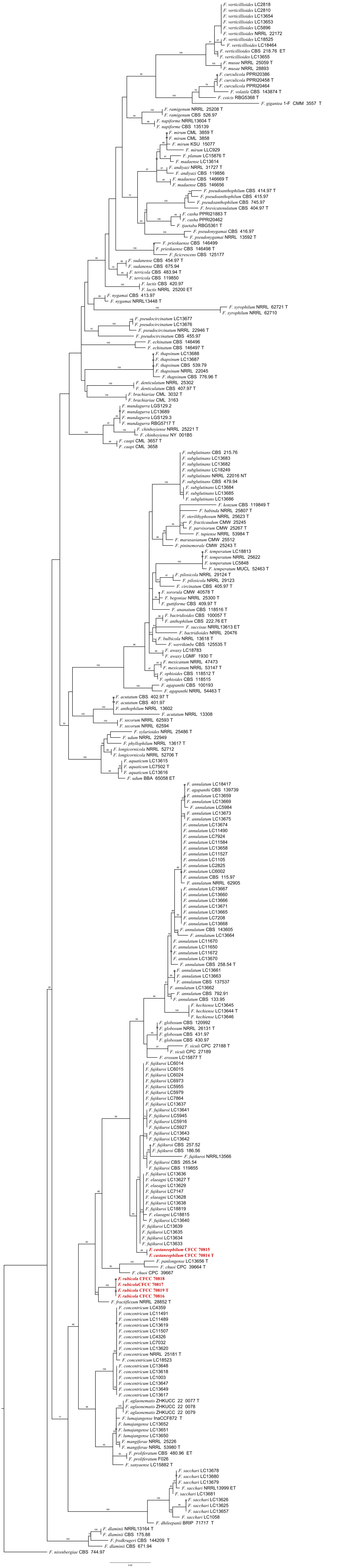
F. verticillioides LC2818
F. verticillioides LC2810
F. verticillioides LC13654
F. verticillioides LC13653
F. verticillioides LC5896
F. verticillioides NRRL 22172
F. verticillioides LC18525
F. verticillioides LC18464
F. verticillioides CBS 218.76 ET
F. verticillioides LC13655
F. musae NRRL 25059 T
F. musae NRRL 28893
F. curculicola PPRI20386
F. curculicola PPRI20458 T
F. curculicola PPRI20464
F. volatile CBS 143874 T
F. coicis RBG5368 T
F. gigantea 1-F CMM 3557 T
F. ramigenum NRRL 25208 T
F. ramigenum CBS 526.97
F. napiforme NRRL13604 T
F. napiforme CBS 135139
F. mirum CML 3859 T
F. mirum CML 3858
F. mirum KSU 15077
F. mirum LLC929
F. planum LC15876 T
F. madaense LC13614
F. andiyazi NRRL 31727 T
F. andiyazi CBS 119856
F. madaense CBS 146669 T
F. madaense CBS 146656
F. pseudoanthophilum CBS 414.97 T
F. pseudoanthophilum CBS 415.97
F. pseudoanthophilum CBS 745.97
F. brevicatenulatum CBS 404.97 T
F. casha PPRI21883 T
F. casha PPRI20462
F. tjaetaba RBG5361 T
F. pseudonygamai CBS 416.97
F. pseudonygamai NRRL 13592 T
F. prieskaense CBS 146499
F. prieskaense CBS 146498 T
F. ficicrescens CBS 125177
F. sudanense CBS 454.97 T
F. sudanense CBS 675.94
F. terricola CBS 483.94 T
F. terricola CBS 119850
F. lactis CBS 420.97
F. lactis NRRL 25200 ET
F. nygamai CBS 413.97
F. nygamai NRRL13448 T
F. xyrophilum NRRL 62721 T
F. xyrophilum NRRL 62710
F. pseudocircinatum LC13677
F. pseudocircinatum LC13676
F. pseudocircinatum NRRL 22946 T
F. pseudocircinatum CBS 455.97
F. echinatum CBS 146496
F. echinatum CBS 146497 T
F. thapsinum LC13688
F. thapsinum LC13687
F. thapsinum CBS 539.79
F. thapsinum NRRL 22045
F. thapsinum CBS 776.96 T
F. denticulatum NRRL 25302
F. denticulatum CBS 407.97 T
F. brachiariae CML 3032 T
F. brachiariae CML 3163
F. mundagurra LGS129.2
F. mundagurra LC13689
F. mundagurra LGS129.3
F. mundagurra RBG5717 T
F. chinhoyiense NRRL 25221 T
F. chinhoyiense NY 001B5
F. caapi CML 3657 T
F. caapi CML 3658
F. subglutinans CBS 215.76
F. subglutinans LC13683
F. subglutinans LC13682
F. subglutinans LC18249
F. subglutinans NRRL 22016 NT
F. subglutinans CBS 479.94
F. subglutinans LC13684
F. subglutinans LC13685
F. subglutinans LC13686
F. konzum CBS 119849 T
F. babinda NRRL 25807 T
F. sterilihyphosum NRRL 25623 T
F. fracticaudum CMW 25245
F. parvisorum CMW 25267 T
F. tupiense NRRL 53984 T
F. marasasianum CMW 25512
F. pininemorale CMW 25243 T
F. temperatum LC18813
F. temperatum NRRL 25622
F. temperatum LC5848
F. temperatum MUCL 52463 T
F. pilosicola NRRL 29124 T
F. pilosicola NRRL 29123
F. circinatum CBS 405.97 T
F. sororula CMW 40578 T
F. begoniae NRRL 25300 T
F. guttiforme CBS 409.97 T
F. ananatum CBS 118516 T
F. bactridioides CBS 100057 T
F. anthophilum CBS 222.76 ET
F. succisae NRRL13613 ET
F. bactridioides NRRL 20476
F. bulbicola NRRL 13618 T
F. werrikimbe CBS 125535 T
F. awaxy LC18783
F. awaxy LGMF 1930 T
F. mexicanum NRRL 47473
F. mexicanum NRRL 53147 T
F. ophioides CBS 118512 T
F. ophioides CBS 118515
F. agapanthi CBS 100193
F. agapanthi NRRL 54463 T
F. acutatum CBS 402.97 T
F. acutatum CBS 401.97
F. anthophilum NRRL 13602
F. acutatum NRRL 13308
F. secorum NRRL 62593 T
F. secorum NRRL 62594
F. xylarioides NRRL 25486 T
F. udum NRRL 22949
F. phyllophilum NRRL 13617 T
F. longicornicola NRRL 52712
F. longicornicola NRRL 52706 T
F. aquaticum LC13615
F. aquaticum LC7502 T
F. aquaticum LC13616
F. udum BBA 65058 ET
F. annulatum LC18417
F. agapanthi CBS 139739
F. annulatum LC13659
F. annulatum LC13669
F. annulatum LC5984
F. annulatum LC13673
F. annulatum LC13675
F. annulatum LC13674
F. annulatum LC11490
F. annulatum LC7924
F. annulatum LC11584
F. annulatum LC13658
F. annulatum LC11527
F. annulatum LC1105
F. annulatum LC2825
F. annulatum LC6002
F. annulatum CBS 115.97
F. annulatum NRRL 62905
F. annulatum LC13667
F. annulatum LC13660
F. annulatum LC13666
F. annulatum LC13671
F. annulatum LC13665
F. annulatum LC7208
F. annulatum LC13668
F. annulatum CBS 143605
F. annulatum LC13664
F. annulatum LC11670
F. annulatum LC11650
F. annulatum LC11672
F. annulatum LC13670
F. annulatum CBS 258.54 T
F. annulatum LC13661
F. annulatum LC13663
F. annulatum CBS 137537
F. annulatum LC13662
F. annulatum CBS 792.91
F. annulatum CBS 133.95
F. hechiense LC13645
F. hechiense LC13644 T
F. hechiense LC13646
F. globosum CBS 120992
F. globosum NRRL 26131 T
F. globosum CBS 431.97
F. globosum CBS 430.97
F. siculi CPC 27188 T
F. siculi CPC 27189
F. erosum LC15877 T
F. fujikuroi LC6014
F. fujikuroi LC6015
F. fujikuroi LC6024
F. fujikuroi LC6973
F. fujikuroi LC5955
F. fujikuroi LC5979
F. fujikuroi LC7864
F. fujikuroi LC13637
F. fujikuroi LC13641
F. fujikuroi LC5945
F. fujikuroi LC5916
F. fujikuroi LC5927
F. fujikuroi LC13643
F. fujikuroi LC13642
F. fujikuroi CBS 257.52
F. fujikuroi CBS 186.56
F. fujikuroi NRRL13566
F. fujikuroi CBS 265.54
F. fujikuroi CBS 119855
F. fujikuroi LC13636
F. elaeagni LC13627 T
F. elaeagni LC13629
F. fujikuroi LC7147
F. elaeagni LC13628
F. fujikuroi LC13638
F. fujikuroi LC18819
F. elaeagni LC18815
F. fujikuroi LC13640
F. fujikuroi LC13639
F. fujikuroi LC13635
F. fujikuroi LC13634
F. fujikuroi LC13633
F. castaneophilum CFCC 70815
F. castaneophilum CFCC 70814 T
F. panlongense LC13656 T
F. chuoi CPC 39664 T
F. chuoi CPC 39667
F. rubicola CFCC 70818
F. rubicolaCFCC 70817
F. rubicola CFCC 70819 T
F. rubicola CFCC 70816
F. fractiflexum NRRL 28852 T
F. concentricum LC4359
F. concentricum LC11491
F. concentricum LC11489
F. concentricum LC13619
F. concentricum LC11507
F. concentricum LC4326
F. concentricum LC7032
F. concentricum LC13620
F. concentricum NRRL 25181 T
F. concentricum LC18523
F. concentricum LC13648
F. concentricum LC13618
F. concentricum LC1003
F. concentricum LC13647
F. concentricum LC13649
F. concentricum LC13617
F. aglaonematis ZHKUCC 22 0077 T
F. aglaonematis ZHKUCC 22 0078
F. aglaonematis ZHKUCC 22 0079
F. lumajangense InaCCF872 T
F. lumajangense LC13652
F. lumajangense LC13651
F. lumajangense LC13650
F. mangiferae NRRL 25226
F. mangiferae NRRL 53980 T
F. proliferatum CBS 480.96 ET
F. proliferatum F026
F. sanyaense LC15882 T
F. sacchari LC13678
F. sacchari LC13680
F. sacchari LC13679
F. sacchari NRRL13999 ET
F. sacchari LC13681
F. sacchari LC13626
F. sacchari LC13625
F. sacchari LC13657
F. sacchari LC1058
F. dhileepanii BRIP 71717 T
F. dlaminii NRRL13164 T
F. dlaminii CBS 175.88
F. fredkrugeri CBS 144209 T
F. dlaminii CBS 671.94
F. nirenbergiae CBS 744.97
0.02
